# Supplementary material for: Effect of power training on physical functional performance of patients with Parkinson’s disease: A systematic review and meta-analysis of randomized controlled trials
Source: PLoS One. 2025 Feb 4;20(2):e0314058. doi: 10.1371/journal.pone.0314058 (PMC11793793; doi:10.1371/journal.pone.0314058)
Supplement: S2 Table — (PDF) [file pone.0314058.s002.pdf]

S2 Table. Search strategy.

Database	Search strategy
Pubmed/MEDLINE	#1: (“exercise” [MeSH] OR physical activity [Title/Abstract] OR “muscle strength” [MeSH] OR physical therapy [Title/Abstract] OR strength training [Title/Abstract] OR strength [Title/Abstract] OR “muscle strength” [MeSH] OR “resistance training” [MeSH] OR power training [Title/Abstract] OR “high speed” [Title/Abstract] OR “high velocity” [Title/Abstract] OR “low resistance” [Title/Abstract]) Field: Title/Abstract<br><br>#2: ((parkinson's disease[MeSH Terms]) OR parkinson[Title/Abstract]) NOT experimental parkinson[Title/Abstract]<br><br>#3: #1 AND #2
Embase	('Parkinson disease':ab, ti OR "Parkinson':ti, ab NOT 'experimental parkinsonism':ab, ti) AND (Exercise:ab, ti OR 'physical activity':ab, ti OR 'muscle strength':ti, ab OR 'physiotherapy':ab, ti OR 'strength training':ab, ti OR 'strength':ab, ti OR 'resistance training':ab, ti OR 'power training':ab, ti)
Lilacs	Descritores: Doença de Parkinson, Força Muscular, Exercício, Fisioterapia
PEDro (Physiotherapy Evidence Database)	Abstract & Title: Parkinson OR Parkinson’s Disease<br><br>Therapy: Strength training<br><br>Method: Clinical Trial
Scopus	TITLE-ABS-KEY (("exercise" OR "physical activity" OR "muscle strength" OR "physical therapy" OR "strength training" OR strength OR "muscle strength" OR "resistance training" OR "power training" OR "high speed" OR "high velocity" OR "low resistance") AND ("parkinson´s disease" OR parkinson))
Cochrane	("Exercise" or "physical activity" or "muscle strength" or "physical therapy" or "strength training" or strength or "muscle strength" or "resistance training" or "power training" or "high speed" or "high velocity" or "low resistance") and ("parkinson´s disease" or parkinson)
