# Supplementary material for: Effect of power training on physical functional performance of patients with Parkinson’s disease: A systematic review and meta-analysis of randomized controlled trials
Source: PLoS One. 2025 Feb 4;20(2):e0314058. doi: 10.1371/journal.pone.0314058 (PMC11793793; doi:10.1371/journal.pone.0314058)
Supplement: S3 Table — (PDF) [file pone.0314058.s003.pdf]

S3 Table. Excluded studies and reasons

Author (Year)	Title	Reason for Exclusion
Agley et al., 2024	Digital Intervention Promoting Physical Activity in People Newly Diagnosed with Parkinson's Disease: Feasibility and Acceptability of the Knowledge, Exercise-Efficacy and Participation (KEEP) Intervention.	Not power training
Ahlskog et al., 2018	Aerobic Exercise: Evidence for a Direct Brain Effect to Slow Parkinson Disease Progression	Not power training
Aitken et al., 2024	Force Control Issues in Upper and Lower Limbs in Parkinson's Disease and Freezing of Gait	Not power training
Akter et al., 2021	Prospective role of polyphenolic compounds in the treatment of neurodegenerative diseases	Not original study
Alberts et al., 2021	Effectiveness of a Long-Term, Home-Based Aerobic Exercise Intervention on Slowing the Progression of Parkinson Disease	Not power training
Alberts et al., 2023	A Randomized Clinical Trial to Evaluate a Digital Therapeutic to Enhance Gait Function in Individuals With Parkinson's Disease	Not power training
Albrecht et al., 2021	Effects of a Highly Challenging Balance Training Program on Motor Function and Brain Structure in Parkinson's Disease	Not power training
Albrecht et al., 2024	Exploring Responsiveness to Highly Challenging Balance and Gait Training in Parkinson's Disease	Not power training
Alcântara et al., 2015	Strength training applied to neurorehabilitation. Physical Exercises: An Important Tool for Physical Therapy	Not original study
Alegre-tamariz et al., 2024	Effects of a sequential square mat walking training	Not power training

S3 Table. Excluded studies and reasons

	program Fisior® on improving physical performance in older adults with Parkinson’s disease: A pilot study.	
Allen et al., 2017	Active arms: a randomized controlled trial of an interactive videogame for people with Parkinson’s disease	Not power training
Allen et al., 2009	Bradykinesia, muscle weakness and reduced muscle power in Parkinson's disease	Insufficient data
Alnes et al., 2023	Mobile health technology, exercise adherence and optimal nutrition post rehabilitation among people with Parkinson’s Disease (mHEXANUT) – a randomized controlled trial protocol	Not power training
Alves et al., 2017	High intensity resistance training improves respiratory, peripheral, quality of life and emotional response in elderly with Parkinson disease	Not power training
Alves et al. (2019)	Strength training improves the respiratory muscle strength and quality of life of elderly with Parkinson disease	Not power training
Ayán et al., 2023	Effects of stretching vs. in Hatha yoga people with mild to moderate Parkinson’s disease: A randomized controlled trial	Not power training
Azizi, et al., 2014	Effects of 8 weeks of water-based exercise on the lower limb muscles strength in Parkinson’s patients	Not power training
Baizabal-carvallo et al., 2023	The Role of Muscle Strength in the Sit-to-Stand Task in Parkinson’s Disease	Not power training
Barbalho et al.,2019	Effects of Low-Volume Resistance Training on Muscle Strength and	Not power training

S3 Table. Excluded studies and reasons

	Functionality of People with Parkinson's Disease	
Barbirato et al., 2013	Muscle strength and executive function as complementary parameters for the assessment of impairment in Parkinson’s disease	Not power training
Barichella et al., 2017	Rehabilitation program with or without a whey protein-based nutritional support enriched with essential aminoacids and vitamin D in patients with Parkinson’s disease	Not power training
Barichella et al., 2018	Rehabilitation program with or without a muscle-targeted nutritional support in patients with Parkinson’s disease or Parkinsonism: study design of a randomized, controlled trial	Not power training
Barichella et al., 2019	Muscle-targeted nutritional support for rehabilitation in patients with parkinsonian syndrome	Not power training
Beck et al., 2018	Can Dual Task Walking Improve in Parkinson’s Disease After External Focus of Attention Exercise?	Not power training
Bega et al., 2015	Yoga versus resistance training in Parkinson’s disease: a 12-week pilot feasibility study	Not original study
Bevilacqua et al., 2020	Rehabilitation of older people with Parkinson’s disease: An innovative protocol for RCT study to evaluate the potential of robotic-based technologies	Not power training
Biebl et al., 2022	Resistance Training Combined with Balance or Gait Training for Patients with Parkinson’s Disease: A Randomized Controlled Pilot Study	Not power training
Bishnoi et al., 2021	Effect of Therapeutic Interventions on Spatiotemporal Gait	Not power training

S3 Table. Excluded studies and reasons

	Parameters in Adults with Neurological Disorders	
Bloomer et al., 2008	Effect of resistance training on blood oxidative stress in Parkinson disease	Not power training
Bloomer et al., 2012	Effect of resistance exercise training on biomarkers of oxidative stress in men and women with Parkinson's disease	Not power training
Bode et al., 2024	Cognition and Activity of Daily Living Function in people with Parkinson's disease.	Not power training
Bolier et al., 2024	Validation of the hotspot for dorsolateral subthalamic nucleus targeting in deep brain stimulation surgery for Parkinson's disease: a post hoc analysis of a randomised controlled trial.	Not power training
Bonde-jensen et al., 2024	Validity and reliability of linear encoder muscle power testing in persons with Parkinson's disease	Not original study
Borders et al., 2023	Motor Performance During Sensorimotor Training for Airway Protection in Parkinson's Disease	Not power training
Borders et al., 2024	Enhancing Cough Motor Learning in Parkinson's Disease Through Variable Practice During Skill Training.	Not power training
Bosch-barceló et al., 2024	A treadmill training program in a gamified virtual reality environment combined with transcranial direct current stimulation in Parkinson's Disease: Study protocol for a randomized controlled trial	Not power training
Brauer s et al., 2017	The effect of a physiotherapy exercise program with a self-management approach vs usual care on physical activity in people with mild-moderate Parkinson's	Not power training

S3 Table. Excluded studies and reasons

	disease: a Randomised Controlled Trial	
Brauer et al., 2024	A physiotherapy group exercise and self-management approach to improve physical activity in people with mild-moderate Parkinson's disease: a randomized controlled trial	Not power training
Bridgewater & sharpe et al., 1997	Trunk muscle training and early Parkinson's disease	Not power training
Brito et al., 2023	Effects of high-intensity respiratory muscle training on respiratory muscle strength in individuals with Parkinson's disease: Protocol of a randomized clinical trial	Not power training
Bryant et al., 2018	Feasibility study: Effect of hand resistance exercise on handwriting in Parkinson's disease and essential tremor	Not original study
Burini et al., 2006	A randomised controlled cross-over trial of aerobic training versus Qigong in advanced Parkinson's disease.	Not power training
Cabrera-martos et al., 2019	A randomized controlled study of whether setting specific goals improves the effectiveness of therapy in people with Parkinson's disease	Not power training
Caglar et al., 2021	Investigation of the Effects of Pilates Training in Parkinson Patients	Not power training
Cakit et al., 2007	The effects of incremental speed-dependent treadmill training on postural instability and fear of falling in Parkinson's disease.	Not power training
Cakmak et al., 2024	Key features of an acceptable trial investigating the effects of dual-task training on balance in people with Parkinson's disease.	Not power training

S3 Table. Excluded studies and reasons

Canning et al., 2012	Minimally-supervised treadmill training for individuals with Parkinson's disease: a randomized controlled trial	Not power training
Canning et al., 2015	Exercise for falls prevention in Parkinson disease	Not power training
Capato et al., 2016	Randomized controlled trial protocol: balance training with rhythmical cues to improve and maintain balance control in Parkinson's disease	Not power training
Carpinella et al., 2017	Wearable Sensor-Based Biofeedback Training for Balance and Gait in Parkinson Disease	Not power training
Carroll et al., 2017	Aquatic Exercise Therapy for People With Parkinson Disease	Not power training
Duplea et al., 2019	Physiological Benefits of High-intensity Interval Training for Individuals With Parkinson's Disease	Not power training
Ceballos-baumann et al., 2018	Non-pharmacological therapies in Parkinsonian syndromes	Not power training
Cedarbaum et al., 1992	Rehabilitation Programs in the Management of Patients with Parkinson's Disease	Not power training
Ceravolo et al., 2009	Rehabilitation goals and strategies in Parkinson's disease	Not power training
Cereda et al., 2022	Whey Protein, Leucine-and Vitamin-D-Enriched Oral Nutritional Supplementation for the Treatment of Sarcopenia	Not power training
Chamberlain-carter et al., 2021	Does resistance training reduce falls and improve quality of life in people with Parkinson's disease using strength training exercise programmes?	Not power training
Chang et al., 2024	Distinct effects of long-term Tai Chi Chuan and aerobic exercise interventions on motor and neurocognitive performance in early-stage	Not power training

S3 Table. Excluded studies and reasons

	Parkinson's disease: a randomized controlled trial	
Chen et al., 2023	The Rehabilitative Effect of Archery Exercise Intervention in Patients with Parkinson's Disease	Not power training
Chen et al., 2024	Assessing the impact of immersive virtual reality technology on the psychological recovery of patients with Parkinson's disease depression: study protocol of a randomized controlled trial	Not original study
Chen et al., 2021	Effects of resistance training on postural control in Parkinson's disease: A randomized controlled trial	Not power training
Chen et al., 2024	The impact of combined aerobic and resistance exercise on the prognosis of early Parkinson's disease patients.	Not power training
Cheng et al., 2016	Positive Effects of Specific Exercise and Novel Turning-based Treadmill Training on Turning Performance in Individuals with Parkinson's disease	Not power training
Cheng et al., 2017	Effects of curved-walking training on curved-walking performance and freezing of gait in individuals with Parkinson's disease	Not power training
Chiong et al., 2019	Effects of physical exercise on gait and balance in patients with Parkinson's disease	Not power training
Choi et al., 2024	Effectiveness and Safety of Meridian Activation Remedy System for Alleviating Motor Symptoms in Parkinson's Disease	Not power training
Choi et al., 2022	Effects of Multimodal Rehabilitation on the Activities of Daily Living, Quality of Life, and Burden of Care for Patients with	Not power training

S3 Table. Excluded studies and reasons

	Parkinson's Disease: A Randomized Control Study	
Chong et al., 2018	A randomized double-blind placebo-controlled trial of probiotics for constipation in Parkinson's disease	Not power training
Chow et al., 2021	The Central Mechanisms of Resistance Training and Its Effects on Cognitive Function	Not power training
Christinelli, et al., 2024	What are the effects of aquatic physiotherapy exercises on speed and complex gait-related activities in individuals with Parkinson's disease?	Not power training
Chung et al., 2010	Baseline measurement and demographics of patients with Parkinson's disease referred for physiotherapy	Not power training
Ciccolo et al., 2013	Resistance training for the prevention and treatment of chronic disease	Not power training
Clael et al., 2023	Cross-education in people with Parkinson's disease, a short-term randomized controlled trial	Not power training
Clael et al., 2019	Strength and physical functions in people with Parkinson's disease	Not power training
Clarke et al., 2016	Physiotherapy and Occupational Therapy vs No Therapy in Mild to Moderate Parkinson Disease: a Randomized Clinical Trial	Not power training
Combs et al., 2013	Community-based group exercise for persons with Parkinson disease: A randomized controlled trial	Not power training
Conradsson et al., 2015	The effects of highly challenging balance training in elderly with Parkinson's disease	Not power training
Corcos et al., 2013	A two-year randomized controlled trial of progressive resistance exercise for Parkinson's disease	Not power training

S3 Table. Excluded studies and reasons

Corcos et al., 2024	Importance of upper and lower body resistance exercise for preventing and reversing sarcopenia in Parkinson's disease	Not power training
Correa et al., 2020	Progressive muscle-strength protocol for the functionality of upper limbs and quality of life in individuals with Parkinson's disease: Pilot study	Not power training
Cox et al., 2016	Thinking in action: Alexander technique for Parkinson's disease	Not power training
Cristini et al., 2024	The Effect of Different Types of Exercise on Sleep Quality and Architecture in Parkinson Disease	Not power training
Da Silva et al., 2023	Comparison of the Effects of an Exergame-Based Program with Conventional Physiotherapy Protocol Based on Core Areas of the European Guideline on Postural Control, Functional Mobility, and Quality of Life in Patients with Parkinson's Disease	Not power training
Dakin et al., 2023	Does Change in Maximal Expiratory Pressure Predict Change in Cough Airflow After Training? A Secondary Analysis of a Randomized Controlled Trial	Not power training
Daniels et al., 2024	Impaired performance of rapid grip in people with Parkinson's disease and motor segmentation	Not power training
David et al., 2012	Progressive resistance exercise and Parkinson's disease: A review of potential mechanisms	Not power training
David et al., 2016	Progressive resistance exercise restores some properties of the triphasic EMG pattern and improves bradykinesia: The PRET-PD randomized clinical trial	Not power training

S3 Table. Excluded studies and reasons

De brito et al., 2023	Effects of high-intensity respiratory muscle training on respiratory muscle strength in individuals with Parkinson's disease	Not power training
De icco et al., 2015	Acute and Chronic Effect of Acoustic and Visual Cues on Gait Training in Parkinson's Disease: A Randomized, Controlled Study	Not power training
De lima et al., 2019	Resistance training reduces depressive symptoms in elderly people with Parkinson disease: A controlled randomized study	Not power training
De moraes et al., 2024	Sensorimotor functioning changes in response to global exercise versus handwriting upper limb exercise training in Parkinson's disease, results from a phase II randomised controlled trial.	Not power training
De Oliveira Braga et al., 2019	EMPOWER-PD - A physical therapy intervention to empower the individuals with Parkinson's disease: A study protocol for a feasibility randomized controlled trial	Not original study
Demonceau et al., 2013	Strength improvement after 3 months of resistance training among Parkinson's disease patients	Not power training
Di cagno et al., 2023	Whole body-electromyostimulation effects on serum biomarkers, physical performances and fatigue in Parkinson's patients: A randomized controlled trial	Not power training
Dibble et al., 2006	High-intensity resistance training amplifies muscle hypertrophy and functional gains in persons with parkinson's disease	Not poqer training
Dibble et al., 2006	The Safety and Feasibility of High-Force Eccentric	Not original study

S3 Table. Excluded studies and reasons

	Resistance Exercise in Persons With Parkinson's Disease	
Dibble et al., 2009	High intensity eccentric resistance training decreases bradykinesia and improves quality of life in persons with Parkinson's disease: A preliminary study	Not power training
Dibble et al., 2015	Exercise and Medication Effects on Persons with Parkinson Disease Across the Domains of Disability: A Randomized Clinical Trial	Not power training
Dietz et al., 1997	Leg muscle activation during gait in Parkinson's disease: Influence of body unloading	Not power training
Dimitrova et al., 2015	Comparison of Two Rehabilitation Programs in Patients With Mild to Moderate Parkinson's Disease	Not power training
Dipasquale et al., 2017	Physical Therapy Versus a General Exercise Programme in Patients with Hoehn Yahr Stage II Parkinson's Disease	Not power training
Dixit et al., 2019	Effectiveness of robotics in improving upper extremity functions among people with neurological dysfunction	Not power training
Domínguez et al., 2016	Effects of resistance training in various pathologies	Not power training
Doruk et al., 2024	Cough and Swallowing Therapy and Their Effects on Vocal Fold Bowing and Laryngeal Lesions	Not power training
Douris et al., 2022	Blood flow restriction resistance training in a recreationally active person with Parkinson's disease	Not power training
Biebl et al., 2022	Resistance Training Combined with Balance or Gait Training for Parkinson's Disease: a	Not power training

S3 Table. Excluded studies and reasons

	Randomized Controlled Pilot Study	
Duarte et al., 2023	Effects of three physical exercise modalities on respiratory function of older adults with Parkinson's disease: A randomized clinical trial	Not power training
Duncan et al., 2023	Does musculoskeletal pain impact physical activity in people with Parkinson disease?	Not power training
Durmus et al., 2010	Lower extremity isokinetic muscle strength in patients with Parkinson's disease	Not power training
Earhart et al., 2015	Comparing interventions and exploring neural mechanisms of exercise in Parkinson disease	Not power training
Ebersbach et al., 2015	Amplitude-oriented exercise in Parkinson's disease: a randomized study comparing LSVT-BIG and a short training protocol	Not power training
El-tamawy et al., 2012	Effects of augmented proprioceptive cues on the parameters of gait of individuals with Parkinson's disease	Not power training
Eldemir et al., 2024	The effects of standard and modified LSVT BIG therapy protocols on balance and gait in Parkinson's disease: A randomized controlled trial	Not power training
Ellis et al., 2005	Efficacy of a physical therapy program in patients with Parkinson's disease: A randomized controlled trial	Not power training
Farley et al., 2005	Training BIG to move faster: the application of the speed-amplitude relation as a rehabilitation strategy for people with Parkinson's disease	Not power training
Feldman et al., 2019	Mindfulness Yoga for managing psychological symptoms of Parkinson's disease	Not power training

S3 Table. Excluded studies and reasons

Silva et al., 2013	Effect of wiireabilitation on trunk mobility of individuals with parkinson disease: A pilot study.	Not power training
Cechetti et al., 2023	Effects of Land and Water Physiotherapy on Motor Function in Parkinson's Disease	Not power training
Fernandes et al., 2020	High-intensity interval versus moderate-intensity continuous training in individuals with Parkinson's disease: Hemodynamic and functional adaptation	Not power training
Ferraz et al., 2017	A comparison of cycle exercise, functional training and Xbox Kinect® in cardiorespiratory fitness of elderly with Parkinson's disease	Not power training
Ferraz et al., 2018	The Effects of Functional Training, Bicycle Exercise, and Exergaming on Walking Capacity of Elderly Patients With Parkinson Disease: A Pilot Randomized Controlled Single-blinded Trial	Not power training
Ferreira et al., 2018	The effect of resistance training on the anxiety symptoms and quality of life in elderly people with parkinson's disease: A randomized controlled trial	Not power training
Fil-balkan et al., 2018	Sensorimotor integration training in Parkinson's disease	Not power training
Fisher et al., 2008	The Effect of Exercise Training in Improving Motor Performance and Corticomotor Excitability in People With Early Parkinson's Disease.	Not power training
Fleisher et al., 2023	Randomized, waitlist-controlled trial of KICK OUT PD: Parkinson's Disease-specific karate yields high	Not power training

S3 Table. Excluded studies and reasons

	adherence and improved quality of life	
Foreman et al., 2012	Exercise and medication induced improvements in postural instability and dynamic balance task performance in persons with Parkinson’s disease	Not power training
Formisano et al., 1992	Rehabilitation and Parkinson’s disease	Not power training
Franzén et al., 2016	Yearly periods of challenging balance training to prevent decline in gait and balance	Not power training
Franzén et al., 2018	Linking neuroplastic effects to behavioral changes after balance training in Parkinson’s disease: A study protocol of a randomized controlled trial	Not power training
Frazzitta et al., 2012	Effectiveness of intensive inpatient rehabilitation treatment on disease progression in parkinsonian patients	Not power training
Frisaldi. Et al., 2021	Effectiveness of a dance-physiotherapy combined intervention in Parkinson’s disease: a randomized controlled pilot trial	Not power training
Gondim et al., 2017	Individualized guidance and telephone monitoring in a self-supervised home-based physiotherapeutic program in Parkinson	Not power training
Fritz et al., 2022	Sensorimotor training at home in elderly people with Parkinson’s Disease during the period of confinement due to COVID-19: A pilot study.	Not power training
Pisano et al., 2014	Three weeks of cycle ergometer versus treadmill training for patients with Parkinson’s disease: a randomized controlled pilot trial.	Not power training
Gwak et al., 2024	Effect of acceptance and commitment therapy on	Not power training

S3 Table. Excluded studies and reasons

	fear of falling and physical activity in Parkinson's disease: A randomised controlled trial.	
Hackney et al., 2020	Rationale and Design of the PAIRED Trial: Partnered Dance Aerobic Exercise as a Neuroprotective, Motor, and Cognitive Intervention in Parkinson's Disease	Not power training
Hallal et al., 2020	Functional training and pilates in individuals with Parkinson's disease	Not power training
Harada et al., 2020	The effects of therapeutic exercise for postural instability in Parkinson's disease: a randomized controlled trial	Not power training
Harris et al., 2024	Gym-Based Modified Powerlifting Exercises for People with Early Onset Parkinson's Disease: Study Protocol.	Not original study
Harvey et al., 2019	High-intensity interval training in people with Parkinson's disease: a randomized, controlled feasibility trial	Not power training
Hass et al., 2012	Progressive resistance training improves gait initiation in individuals with Parkinson's disease	Not power training
Hass et al., 2007	Resistance training with creatine monohydrate improves upper-body strength in patients with Parkinson disease: A randomized trial	Not power training
Helgerud et al., 2020	Maximal strength training in patients with Parkinson's disease: Impact on efferent neural drive, force-generating capacity, and functional performance	Not power training
Hewitt et al., 2018	Progressive Resistance and Balance Training for Falls Prevention in Long-Term Residential Aged Care: A	Not power training

S3 Table. Excluded studies and reasons

	Cluster Randomized Trial of the Sunbeam Program	
Hirsch et al., 2003	The effects of balance training and high-intensity resistance training on persons with idiopathic Parkinson's disease	Not power training
Hoffman et al., 2023	Efficacy and Feasibility of Remote Cognitive Remediation Therapy in Parkinson's Disease: A Randomized Controlled Trial	Not power training
Hortobágyi et al., 2022	The impact of aerobic and resistance training intensity on markers of neuroplasticity in health and disease	Not power training
Hubble et al., 2018	Trunk Exercises Improve Gait Symmetry in Parkinson Disease: A Blind Phase II Randomized Controlled Trial	Not power training
Hulbert et al., 2019	"PDSAFE'-a multi-dimensional model of falls rehabilitation for people with Parkinson's. A mixed methods analysis of therapists" delivery and experience	Not power training
Intzandt et al., 2015	Two Resistance Training Protocols to Reduce the Risk of Falls in Parkinson's Disease	Not power training
Oliveira-filho et al., 2019	Study of the Effects of Three Exercise Programs in Parkinson's Disease	Not power training
Jaywant et al., 2016	Randomized Controlled Trial of a Home-Based Action Observation Intervention to Improve Walking in Parkinson Disease	Not power training
Johnson et al., 2021	Feasibility and tolerability randomized clinical trial of golf versus Tai Chi for people with moderate Parkinson's Disease	Not original study

S3 Table. Excluded studies and reasons

Joseph et al., 2018	Implementation of the HiBalance training program for Parkinson's disease in clinical settings	Not power training
Jung et al., 2020	Effects of the agility boot camp with cognitive challenge (ABC-C) exercise program for Parkinson's disease	Not power training
Kadkhodaie et al., 2020	Effect of eccentric-based rehabilitation on hand tremor intensity in Parkinson disease	Not power training
Kakinuma et al., 1998	Muscle weakness in Parkinson's disease: Isokinetic study of the lower limbs	Not power training
Kanegusuku et al., 2017	Effects of Progressive Resistance Training on Cardiovascular Autonomic Regulation in Patients With Parkinson Disease: A Randomized Controlled Trial	Not power training
Kargarfard et al., 2012	Effects of an 8-week aquatic exercise training on balance in patients with Parkinson's disease	Not power training
Kathia et al., 2024	High-intensity interval versus moderate-intensity continuous cycling training in Parkinson's disease: a randomized trial.	Not power training
Aytutuldu, et al., 2024	LSVT® BIG versus progressive structured mobility training through synchronous telerehabilitation in Parkinson's disease: A randomized controlled trial	Not power training
Kelly et al., 2018	Effects of aging and Parkinson's disease on motor unit remodeling: Influence of resistance exercise training	Not power training
Koli et al., 2018	Indian semi-classical kathak and bharatnatyam movements for balance	Not power training

S3 Table. Excluded studies and reasons

	confidence and quality of life in Parkinson's disease	
Kottaras et al., 2021	Effect of aquatic physiotherapy on functioning, balance performance, motor performance, and health-related quality of life in patients with parkinson's disease	Not power training
Kuo et al., 2017	Effect of expiratory muscle strength training intervention on the maximum expiratory pressure and quality of life of patients with Parkinson disease	Not power training
Kurt et al., 2018	Effects of Ai Chi on balance, quality of life, functional mobility, and motor impairment in patients with Parkinson's disease	Not power training
Kurtais et al., 2008	Does treadmill training improve lower-extremity tasks in Parkinson disease?	Not power training
Kwok et al., 2017	The effects of yoga versus stretching and resistance training exercises on psychological distress for people with Parkinson's disease	Not power training
Kwok et al., 2019	Effects of Mindfulness Yoga vs Stretching and Resistance Training Exercises on Anxiety and Depression for People with Parkinson Disease: A Randomized Clinical Trial	Not power training
Kwok et al., 2023	A randomized clinical trial of mindfulness meditation versus exercise in Parkinson's disease during social unrest	Not power training
Kwok et al., 2017	The effects of yoga versus stretching and resistance training exercises on psychological distress for people with mild-to-moderate Parkinson's	Not original study

S3 Table. Excluded studies and reasons

	disease: Study protocol for a randomized controlled trial	
Lang et al., 2019	Antagonist muscle activity during reactive balance responses is elevated in Parkinson's disease and in balance impairment	Not power training
Langbroek-amersfoort et al., 2023	Exercise Made Accessible: the Merits of Community-Based Programs for Persons with Parkinson's Disease	Not power training
Langer et al., 2024	Vertical locomotion improves horizontal locomotion: effects of climbing on gait and other mobility aspects in Parkinson's disease. A secondary analysis from a randomized controlled trial	Not power training
Langer et al., 2023	Climb up! Head up! Climbing improves posture in Parkinson's disease. A secondary analysis from a randomized controlled trial	Not power training
Leal et al., 2019	Low-volume resistance training improves the functional capacity of older individuals with Parkinson's disease	Not power training
Leavy, b. Et al., 2023	Design of the STEPS trial: a phase II randomized controlled trial evaluating eHealth-supported motor-cognitive home training for Parkinson's disease	Not power training
Li et al., 2012	Postural Stability in Parkinson'S Disease Patients After Tai Chi Training: a Randomized Controlled Trial	Not power training
Li et al., 2024	The effects of Baduanjin on fine motor skills in mild and moderate Parkinson's disease: A randomized controlled trial	Not power training
Li et al., 2024	Adherence to Non-Pharmacological Interventions in Parkinson's Disease: A Rapid Evidence	Not power training

S3 Table. Excluded studies and reasons

	Assessment of the Literature.	
Lima et al., 2013	Recruitment rate, feasibility and safety of power training in individuals with Parkinson's disease: A proof-of-concept study	Not original study
Lima et al., 2019	Resistance training reduces depressive symptoms in elderly people with Parkinson disease: a controlled randomized study	Not power training
Lima et al., 2020	Effects of a power strength training using elastic resistance exercises on the motor and non-motor symptoms in patients with Parkinson's disease H&Y 1-3: study protocol for a randomised controlled trial (PARK-BAND Study)	Not power training
Lima et al., 2021	PARK-BAND training program for Parkinson's disease in clinical settings: a feasibility study	Not original study
Lima et al., 2012	Muscular power training: A new perspective in physical therapy approach of Parkinson's disease	Not original study
Lopane et al., 2022	Long-term wearable tele-rehabilitation of gait for persons with Parkinson's Disease: A feasibility study.	Not original study
Loureiro et al., 2021	Efficacy of Rehabilitation on Mobility, Prevention and Reduction of the Fall's Risk in Parkinson's Patients	Not power training
Luo et al., 2024	Effectiveness of Yijinjing on cognitive and motor functions in patients with Parkinson's disease: study protocol for a randomized controlled trial	Not power training
Mahalakshmi et al., 2020	Possible neuroprotective mechanisms of physical exercise in neurodegeneration	Not power training

S3 Table. Excluded studies and reasons

Mahmoud et al., 2018	Efficacy of neurofunctional versus resistance training in improving gait and quality of life among patients with Parkinson's disease: A randomized clinical trial	Not power training
Mak et al., 2008	Cued task-specific training is better than exercise in improving sit-to-stand in patients with Parkinson's disease	Not power training
Mak et al., 2022	Feasibility of using mobile technology for aerobic exercise management in people with Parkinson's Disease	Not power training
Mak et al., 2024	Effectiveness of Balance Exercise and Brisk Walking on Alleviating Nonmotor and Motor Symptoms in People With Parkinson Disease	Not power training
Mak et al., 2023	Long-term effects of a 6-month brisk walking and balance program on physical performance and health-related quality of life in people with Parkinson disease: A randomized controlled trial	Not power training
Malin et al., 2021	Power Training for Improvement of Postural Stability and Reduction of Falls in Individuals with Parkinson Disease.	Not power training
Manca et al., 2019	Isokinetic predictors of gait speed increase following high-intensity resistance training of the ankle dorsiflexors in people with multiple sclerosis: A pilot study	Not power training
Mansfield et al., 2015	Perturbation training to promote safe independent mobility post-stroke	Not power training
Barboza, et al., 2019	Physiotherapy Versus Physiotherapy Plus Cognitive Training on Cognition and Quality of	Not power training

S3 Table. Excluded studies and reasons

	Life in Parkinson Disease: randomized Clinical Trial	
Martins et al., 2018	Resistance Training in Patients With Parkinson's Disease	Not power training
Mateos-toset et al., 2016	Effects of a Single Hand-Exercise Session on Manual Dexterity and Strength in Persons with Parkinson Disease	Not power training
Mcgibbon et al., 2024	Exercising with a robotic exoskeleton can improve memory and gait in people with Parkinson's disease	Not power training
Mcginley et al., 2012	Feasibility, safety, and compliance in a randomized controlled trial of physical therapy for parkinson's disease	Not power training
Meimandi et al., 2023	A comparison of the effects of occupation-based interventions with and without responsibility feedback and conventional interventions on participation in people with idiopathic Parkinson's disease: study protocol for a randomized controlled trial	Not power training
Menacho et al., 2022	Effects of Physical Exercise in movements, manual hability and brain plasticity in individuals with Parkinson's disease	Not power training
Menacho et al., 2023	Exercise effects on motor function, manual dexterity, and brain oscillatory activity in individuals with Parkinson's disease: Randomized controlled trial protocol	Not power training
Meng ni et al., 2016	Power training induced change in bradykinesia and muscle power in Parkinson's disease	Insufficient data
Meng ni et al., 2017	High-speed resistance training modifies load-velocity and load-power	Insufficient data

S3 Table. Excluded studies and reasons

	relationships in Parkinson's disease	
Mildner et al., 2024	Effects of activity-oriented physiotherapy with and without eye movement training on dynamic balance, functional mobility, and eye movements in patients with Parkinson's disease: An assessor-blinded randomised controlled pilot trial.	Not power training
Miyasato et al., 2018	Cardiovascular Responses During Resistance Exercise in Patients With Parkinson Disease	Not power training
Mohammadpour et al., 2018	Effects of a combined aerobic and resistance exercise program on the quality of life and motor function of elderly men with Parkinson's disease	Not power training
Monleon et al., 2023	Functional balance training in people with Parkinson's disease: a protocol of balanceHOME randomized control trial with crossover	Not power training
Montero et al., 2019	Effects of inspiratory muscle training on respiratory muscle strength, lung function, functional capacity and cardiac autonomic function in Parkinson's disease: Randomized controlled clinical trial protocol	Not power training
Monticone et al., 2015	In-patient multidisciplinary rehabilitation for Parkinson's disease	Not original study
Moon et al., 2024	A pilot randomized clinical trial examining the effects of Qigong on inflammatory status and sleep quality in people with Parkinson's disease	Not power training
Moraca et al., 2024	Aerobic exercise on the treadmill combined with transcranial direct current stimulation on the gait of	Not original study

S3 Table. Excluded studies and reasons

	people with Parkinson's disease: A protocol for a randomized clinical trial.	
Morberg et al., 2014	The impact of high intensity physical training on motor and non-motor symptoms in patients with Parkinson's disease (PIP): A preliminary study	Not power training
Morley et al., 2022	Effect of exercise on motor symptoms, non-motor symptoms and dopamine transporter imaging in Parkinson Disease	Not power training
Moroz et al., 2009	Rehabilitation Interventions in Parkinson Disease	Not power training
Morris et al., 2009	A randomized controlled trial of movement strategies compared with exercise for people with Parkinson's disease	Not power training
Morris et al., 2012	Protocol for a home-based integrated physical therapy program to reduce falls and improve mobility in people with Parkinson's disease	Not power training
Morris et al., 2013	What are the best methods to prevent falls in Parkinson's disease?	Not power training
Morris et al., 2015	A Randomized Controlled Trial to Reduce Falls in People with Parkinson's Disease	Not power training
Morris et al., 2017	A home program of strength training, movement strategy training and education did not prevent falls in people with Parkinson's disease: a randomised trial	Not power training
Munzert et al., 2009	Cognitive motor processes: The role of motor imagery in the study of motor representations	Not power training
Naqvi et al., 2022	Comparative effects of high and low intensity resistance training on balance and fatigue perception in	Not power training

S3 Table. Excluded studies and reasons

	patients with Parkinson's Disease	
Nascimento et al., 2022	Phase angle, muscle mass, and functionality in patients with Parkinson's disease	Not power training
Ni et al., 2016	Controlled pilot study of the effects of power yoga in Parkinson's disease	Not power training
Nogaki et al., 1995	Muscle strength in early Parkinson's disease	Not power training
Nogaki et al., 1999	Movement velocity dependent muscle strength in Parkinson's disease	Not power training
Nogaki et al., 2001	Muscle weakness in Parkinson's disease: A follow-up study	Not power training
Ortiz-rubio et al., 2018	Effects of a resistance training program on balance and fatigue perception in patients with Parkinson's disease: A randomized controlled trial	Not power training
O'brien et al., 2008	A qualitative analysis of a progressive resistance exercise programme for people with Parkinson's disease	Not power training
Palamara et al., 2017	Land Plus Aquatic Therapy Versus Land-Based Rehabilitation Alone for the Treatment of Balance Dysfunction in Parkinson Disease: A Randomized Controlled Study With 6-Month Follow-Up	Not power training
Pang & mak et al., 2012	Influence of contraction type, speed, and joint angle on ankle muscle weakness in Parkinson's disease: Implications for rehabilitation	Not power training
Pang et al., 2009	Trunk muscle strength, but not trunk rigidity, is independently associated with bone mineral density of the lumbar spine in patients with Parkinson's disease	Not power training

S3 Table. Excluded studies and reasons

Paolucci et al., 2017	Impact of Mézières Rehabilitative Method in Patients with Parkinson's Disease	Not power training
Paramasivam & kumar, 2020	Efficacy of aquatic therapy in rehabilitation of balance, gait & lower extremity function in Parkinson's disease - a randomized clinical trial	Not power training
Park et al., 2024	The efficacy and safety of MARS-PD: Meridian activation remedy system for Parkinson's disease—A single-center, assessor and statistician-blinded, parallel-group randomized, controlled trial protocol	Not power training
Pastana et al., 2023	Feasibility and effectiveness of a remote individual rehabilitation program for people with Parkinson's disease living in the Brazilian Amazon: a randomized clinical trial	Not power training
Paul et al., 2013	Leg muscle power training in Parkinson's disease: a randomised controlled trial	Not power training
Paul et al., 2011	Muscle power training in Parkinson's disease	Not power training
Peek et al., 2016	Resistance training for people with Parkinson's disease (PEDro synthesis)	Not power training
Peters et al., 2012	A randomized controlled trial of an enhanced interdisciplinary community based group program for people with parkinson's disease	Not power training
Picelli et al., 2015	Rehabilitation procedures in the management of Parkinson's disease	Not power training
Pinto et al., 2024	Online dance versus online therapeutic exercise on quality of life - a study protocol for a randomized controlled trial investigating social	Not original study

S3 Table. Excluded studies and reasons

	telerehabilitation efficacy in Parkinson disease	
Podlewska et al., 2024	The PD-Ballet study: study protocol for a randomised controlled single-blind hybrid type 2 clinical trial evaluating the effects of ballet dancing on motor and non-motor symptoms in Parkinson's disease	Not original study
Pretzer-aboff, et al., 2010	Long term impact of a restorative care intervention for people with Parkinson's in the community setting.	Not power training
Prodoehl et al., 2015	Two-year exercise program improves physical function in Parkinson's disease: The PRET-PD randomized clinical trial	Not power training
Pélissier & pérennou et al., 2000	Exercices program and rehabilitation of motor disorders in Parkinson's disease	Not power training
Raccagni et al., 2024	Effects of physiotherapy and home-based training in parkinsonian syndromes: protocol for a randomised controlled trial (MobilityAPP).	Not power training
Rafferty et al., 2012	Long-term exercise improves and maintains physical function in people with Parkinson's disease	Not power training
Rafferty et al., 2017	Effects of 2 years of exercise on gait impairment in people with Parkinson Disease: The PRET-PD randomized trial	Not power training
Ribas et al., 2017	Effectiveness of exergaming in improving functional balance, fatigue and quality of life in Parkinson's disease	Not power training
Rick et al., 2010	PD REHAB: Randomized controlled trial to study the effectiveness and cost-effectiveness of physiotherapy and	Not power training

S3 Table. Excluded studies and reasons

	Occupational therapy for people with PD	
Romanato et al., 2020	Muscle forces and activations in Parkinson's disease: a model-based approach	Not power training
Rosales et al., 2015	Effect of a training method of resistance against the functional capacity and quality of life of subjects with Parkinson idiopathic	Not power training
Rotondo et al., 2024	Dose–response effects of physical exercise standardized volume on peripheral biomarkers, clinical response, and brain connectivity in Parkinson's disease	Not power training
Valenzuela et al., 2020	Effects of Dual-Task Group Training on Gait, Cognitive Executive Function, and Quality of Life in People With Parkinson Disease: Results of Randomized Controlled DUALGAIT Trial	Not power training
Santos et al., 2017	Effects of progressive resistance exercise in akinetic-rigid Parkinson's disease patients: a randomized controlled trial	Not power training
Santos et al., 2017	Balance versus resistance training on postural control in patients with Parkinson's disease: A randomized controlled trial	Not power training
Santos et al., 2021	Being physically active minimizes the effects of leg muscle fatigue on obstacle negotiation in people with Parkinson's disease	Not power training
Santos et al., 2018	Strength Training and Different Subtypes of Parkinson´s Disease	Not power training
Sapienza et al., 2012	Strength training outcomes for airway protection in PD	Not power training
Sarasso et al., 2024	Neural correlates of bradykinesia in Parkinson's disease: a kinematic and functional MRI study.	Not power training

S3 Table. Excluded studies and reasons

Scandalis et al., 2001	Resistance training and gait function in patients with Parkinson's disease	Not power training
Schenkman et al., 2010	A randomized controlled trial of movement strategies compared with exercise for people with Parkinson's disease	Not power training
Schenkman et al., 2012	Exercise for people in early- or mid-stage parkinson disease: A 16-month randomized controlled trial	Not power training
Schenkman et al., 1998	Exercise to improve spinal flexibility and function for people with Parkinson's disease: a randomized, controlled trial	Not power training
Schilling & hammond et al., 2013	Resistance training for Parkinson's disease	Not power training
Schilling et al., 2010	Effects of moderate-volume, high-load lower-body resistance training on strength and function in persons with Parkinson's disease: a pilot study	Not power training
Schlenstedt et al., 2015	Resistance versus balance training to improve postural control in Parkinson's disease: A randomized rater blinded controlled study	Not power training
Schlenstedt et al., 2018	Moderate Frequency Resistance and Balance Training Do Not Improve Freezing of Gait in Parkinson's Disease: A Pilot Study	Not power training
Schmidt et al., 2018	How accurate are exercise logbooks? A comparison of participant and device recorded exercise records in people with Parkinson's disease	Not power training
Schootemeijer et al., 2022	The STEPWISE Parkinson study: a smartphone-based exercise solution for patients with Parkinson's disease	Not power training

S3 Table. Excluded studies and reasons

Seel & cifu et al., 2005	Rehabilitation and neurologic repair in Parkinson's disease	Not Parkinson's disease
Shabanpour et al., 2022	Parkinson's disease is characterized by sub-second resting-state spatio-oscillatory patterns: A contribution from deep convolutional neural network	Not power training
Shen et al., 2014	Balance and gait training with augmented feedback improves balance confidence in people with parkinson's disease	Not power training
Shulman et al., 2013	Randomized clinical trial of 3 types of physical exercise for patients with parkinson disease	Not power training
Sidoroff et al., 2022	Gait and Balance Characteristics in Atypical Parkinsonian Syndromes	Not power training
Siega et al., 2021	Parkinson's disease and multicomponent aquatic exercise: Effects on motor aspects, functional mobility, muscle function and aquatic motor skills	Not power training
Siegert et al., 2019	ParkProTrain: An individualized, tablet-based physiotherapy training programme aimed at improving quality of life and participation restrictions in PD patients - A study protocol for a quasi-randomized, longitudinal and sequential multi-method study	Not power training
Signorile et al., 2017	Comparative time course changes between Parkinson's disease and age-matched elders during power resistance training	Not power training
Signorile et al., 2022	Mindfulness and Yoga or Resistance Exercise Training For Parkinson's Patients	Not power training

S3 Table. Excluded studies and reasons

Silva et al., 2024	Immersive virtual reality as a complement to physical therapy for Parkinson's disease – A randomized controlled trial	Not power training
Silva-batista et al., 2016	Resistance Training with Instability for Patients with Parkinson's Disease	Not power training
Silva-batista et al., 2017	Resistance training improves sleep quality in subjects with moderate Parkinson's disease	Not power training
Silva-batista et al., 2017	Resistance training with instability is more effective than resistance training in improving spinal inhibitory mechanisms in Parkinson's disease	Not power training
Silva-batista et al., 2018	Balance and fear of falling in subjects with Parkinson's disease is improved after exercises with motor complexity	Not power training
Silva-batista et al., 2019	Short-term resistance training with instability reduces impairment in V wave and H reflex in individuals with Parkinson's disease	Not power training
Silva-batista et al., 2023	Balance telerehabilitation and wearable technology for people with Parkinson's disease (TelePD trial)	Not power training
Smaili et al., 2018	Efficacy of neurofunctional versus resistance training in improving gait and quality of life among patients with Parkinson's disease: A randomized clinical trial	Not power training
Smart et al., 2020	Importance of maximal strength and muscle-tendon mechanics for improving force steadiness in persons with Parkinson's disease	Not power training
Song et al., 2017	Home-based step training using videogame technology in people with Parkinson's disease	Not power training

S3 Table. Excluded studies and reasons

Sousa et al., 2023	Strength Training for patients with Parkinson's Disease identified by a biochemical examination	Not power training
Souza et al., 2020	Resistance training on postural control in Parkinson's Disease: a randomized controlled trial	Not power training
Speelman et al., 2010	Design of a randomized controlled trial evaluating the effectiveness of a multifaceted program to increase physical activity in Parkinson patients	Not power training
Speelman et al., 2010	Rationale and design of the ParkFit study: A randomized controlled trial to increase physical activity in patients with Parkinson's disease	Not power training
Steube et al., 2011	Rehabilitation of Parkinson's disease - The LSVT® BIG/LSVT® LOUD concept	Not power training
Steurer et al., 2024	Using Portable Voice Accumulators to Study Transfer of Speech Outcomes Following Intervention	Not power training
Stevens-lapsley et al., 2012	Quadriceps muscle weakness, activation deficits, and fatigue with parkinson Disease	Not power training
Stożek et al., 2016	The effect of the rehabilitation program on balance, gait, physical performance and trunk rotation in Parkinson's disease	Not power training
Strand et al., 2021	Periodized Resistance Training with and without Functional Training Improves Functional Capacity, Balance, and Strength in Parkinson's Disease	Not power training
Stuckenschneider et al., 2015	Active assistive forced exercise provides long-term improvement to gait	Not power training

S3 Table. Excluded studies and reasons

	velocity and stride length in patients bilaterally affected by Parkinson's disease	
Szu-min et al., 2021	Interactive POWER Rehabilitation System on Rehabilitation Training for Patients With Parkinson's Disease	Not power training
Tanović et al., 2019	Effects of kinesiotherapy on muscle strengthening in patients with Parkinson disease	Not power training
Troche et al., 2023	Rehabilitating Cough Dysfunction in Parkinson’s Disease: A Randomized Controlled Trial	Not power training
Tsang et al., 2013	Tai Chi training is effective in reducing balance impairments and falls in patients with Parkinson's disease	Not power training
Tunur et al., 2020	Augmented reality-based dance intervention for individuals with Parkinson’s disease: A pilot study	Not power training
Ugrinowitsch et al., 2013	Functional and strength training in parkinsonians	Not power training
Uygur et al., 2017	Effects of a low-resistance, interval bicycling intervention in Parkinson’s Disease	Not power training
Van der kolk et al., 2015	Design of the Park-in-Shape study: A phase II double blind randomized controlled trial evaluating the effects of exercise on motor and non-motor symptoms in Parkinson’s disease	Not power training
Van nimwegen et al., 2010	Monitoring treatment fidelity in the ParkFit study, a multifaceted behavioral program to increase physical activity in Parkinson patients.	Not power training
Vanbellingen et al., 2017	Home based training for dexterity in Parkinson’s disease	Not power training

S3 Table. Excluded studies and reasons

Vasconcellos et al., 2023	Telerehabilitation-based trunk exercise training for motor symptoms of individuals with Parkinson's disease: A randomized controlled clinical trial	Not power training
De moraes filho et al., 2020	Progressive Resistance Training Improves Bradykinesia, Motor Symptoms and Functional Performance in Patients with Parkinson's Disease	Not power training
Vieira-yano et al., 2021	The Adapted Resistance Training with Instability Randomized Controlled Trial for Gait Automaticity	Not power training
Wang et al., 2018	Influence of rhythmic auditory attention stimulation combined with intensive muscle strength training on lower limb function and balance function in elderly patients with Parkinson's disease	Not power training
Wang et al., 2021	Long-Term Wu Qin Xi Exercise on Response Inhibition and Cortical Connectivity in Parkinson's Disease	Not power training
Wang et al., 2023	Effect of Progressive Postural Control Exercise Versus Core Stability Exercise in Young Adults with Chronic Low Back Pain: A Randomized Controlled Trial	Not power training
Wu et al., 2021	Effects of home-based exercise on motor, non-motor symptoms and health-related quality of life in Parkinson's disease patients: A randomized controlled trial	Not power training
Xu et al., 2024	Effects of ankle isokinetic training on muscle strength and balance amongst older women with mild	Insufficient data

S3 Table. Excluded studies and reasons

	Parkinson's disease: A randomized trial	
Ya-yun et al., 2022	Benefits of Different Exercise Types to Enhance Walking for PD	Not power training
Yaghoob et al., 2019	Comparison of the effect of aerobic and resistance training on cardiovascular risk factors in Parkinson's women	Not power training
Yan et al., 2024	The effect of multi-component exercise intervention in older people with Parkinson's disease and mild cognitive impairment: A randomized controlled study.	Not power training
Ying et al., 2021	Study on the rehabilitation effect of low-load exercise training on motor function and quality of life of patients with Parkinson's disease	Not power training
Yokote et al., 2022	Leg Muscle Strength Correlates with Gait Performance in Advanced Parkinson Disease	Not power training
Youm et al., 2020	Impact of trunk resistance and stretching exercise on fall-related factors in patients with Parkinson's disease: A randomized controlled pilot study	Not power training
Yu et al., 2024	Evidence map of traditional Chinese exercises.	Not power training
Yuen et al., 2019	The effect of conduction exercise and self-acupressure in treatment of Parkinson's disease: Protocol for a pilot study	Not power training
Zare et al., 2022	Effect of activity-based training versus strengthening exercises on upper extremity functions in Parkinson's patients; A randomized controlled, single blind, superiority trial	Not power training

S3 Table. Excluded studies and reasons

Zhen et al., 2022	Effects of Wu Qin Xi exercise on reactive inhibition in Parkinson's disease: a randomized controlled clinical trial	Not power training
Zhu et al., 2021	Reliability check of an assessment system for Parkinson's disease tremor monitoring with portable devices	Not power training
Ziegler et al., 2018	Falls in patients with Parkinson's disease syndromes and their prevention with physiotherapy	Not power training
Brito et al., 2023	Effects of high-intensity respiratory muscle training on respiratory muscle strength in individuals with Parkinson's disease: Protocol of a randomized clinical trial	Not power training
Khobkhun et al., 2021	The effects of a 10-week home-based exercise programme in individuals with Parkinson's disease during the covid-19 pandemic: A pilot study	Not power training
Ayán et al., 2012	Effects of aquatic exercise on persons with Parkinson's disease: A preliminary study	Not power training
Geroin et al., 2015	The effects of different gait training techniques for improving walking performance in patients in the early stages of Parkinson's Disease: a pilot randomized clinical trial	Not power training
Kim et al., 2023	Effects of high-intensity interval training and moderate-intensity continuous training on sarcopenia-related parameters in participants with Parkinson's disease: A 24-week randomized pilot trial substudy	Not power training

S3 Table. Excluded studies and reasons

Dönmez et al., 2010	The effects of home exercise program on balance and functional capacity in parkinsonian patients	Not power training
Smimih et al., 2023	Effects of physical activity on Pakinson's disease patients. Experimental and Clinical Evidence of the Neuropathology of Parkinson's Disease	Not power training
Lee et al., 2019	Effects of virtual reality exercise program using the sony playstation 2 gaming platform on balance, emotion and quality of life in patients with Parkinson's disease	Not power training
King et al., 2015	Effects of group, individual, and home exercise in persons with Parkinson disease: A randomized clinical trial	Not power training
Sohmiya et al., 2013	Immediate effects of physical therapy on gait disturbance and frontal assessment battery in Parkinson's disease	Not power training
Kaseda et al., 2017	Therapeutic effects of intensive inpatient rehabilitation in advanced Parkinson's disease	Not power training
Plaza et al., 2022	Effects of a tongue training program in Parkinson's disease: Analysis of electrical activity and strength of suprahyoid muscles	Not power training
Lun et al., 2005	Comparison of the effects of a self-supervised home exercise program with a physiotherapist-supervised exercise program on the motor symptoms of Parkinson's disease	Not power training
Filippin et al., 2017	Treadmill training with additional body load: Effects on the gait of people with parkinson's disease	Not power training

S3 Table. Excluded studies and reasons

Ekmekyapar et al., 2023	Effects of LSVT-BIG via telerehabilitation on non-motor and motor symptoms and quality of life in Parkinson's disease	Not power training
Ferraz et al., 2018	The Effects of Functional Training, Bicycle Exercise, and Exergaming on Walking Capacity of Elderly Patients With Parkinson Disease: A Pilot Randomized Controlled Single-blinded Trial	Not power training
Kargarfard et al., 2012	Effects of an 8-week aquatic exercise training on balance in patients with Parkinson's disease	Not power training
Ortiz-rubio et al., 2018	Effects of a resistance training program on balance and fatigue perception in patients with Parkinson's disease: A randomized controlled trial	Not power training
Zhang et al., 2015	Effects of Tai Chi and multimodal exercise training on movement and balance function in mild to moderate idiopathic Parkinson disease	Not power training
Vialleron et al., 2021	Acute effects of short-term stretching of the triceps surae on ankle mobility and gait initiation in patients with Parkinson's disease	Not power training
Kaya et al., 2023	The effects of telerehabilitation-based LSVT-BIG versus telerehabilitation-based structured balance and mobility exercise Program on functional recovery in patients with Parkinson's disease	Not power training
Amato et al., 2022	Effects of a Resistance Training Protocol on Physical Performance, Body Composition, Bone Metabolism, and Systemic	Not power training

S3 Table. Excluded studies and reasons

	Homeostasis in Patients Diagnosed with Parkinson's Disease: A Pilot Study	
Sohng et al., 2007	The development and effects of a self-management program for patients with Parkinson's disease	Not power training
Jäggi et al., 2023	Feasibility and effects of cognitive–motor exergames on fall risk factors in typical and atypical Parkinson's inpatients: a randomized controlled pilot study	Not power training
Lazaro et al., 2011	The immediate effects of torso weighting on balance and mobility of persons with parkinson's disease	Not power training
Domínguez et al., 2016	Effects of resistance training in various pathologies	Not power training
Svensson et al., 2015	Effects of Physical Exercise on Neuroinflammation, Neuroplasticity, Neurodegeneration, and Behavior	Not power training
Wu et al., 2021	Effects of Aerobic Exercise and Mind-Body Exercise in Parkinson's Disease: A Mixed-Treatment Comparison Analysis	Not power training
Laupheimer et al., 2013	Effects of MOTOmed® exercise on typical motor dysfunction in Parkinson's disease	Not power training
Nocera et al., 2009	Effects of home-based exercise on postural control and sensory organization in individuals with Parkinson disease	Not power training
Gomez-lopez et al., 2023	Effects of a Multicomponent Physical Exercise Program on the Physiological Functions of People With Parkinson's Disease	Not power training
Sangarapillai et al., 2020	Are effects of Rock Steady Boxing and PD SAFExTM on the motor symptoms of	Not power training

S3 Table. Excluded studies and reasons

	Parkinson's disease maintained long-term?	
Köseoğlu et al., 1997	Effects of a pulmonary rehabilitation program on pulmonary function in Parkinson's disease	Not power training
Caglar et al., 2005	Effects of home exercises on motor performance in patients with Parkinson's disease	Not power training
Jenks et al., 2019	Effects of intensive exercise-based swallowing program for Parkinson's disease	Not power training
Smulders et al., 2015	Effects of exercise on mobility, cognition and locomotor circuit connectivity in Parkinsonism	Not power training
Lee et al., 2019	Effects of virtual reality exercise therapy on balance function and quality of life in Parkinson's disease	Not power training
Johnson et al., 2016	Effects of a 12-week exercise program on cognition in Parkinson's disease	Not power training
Güngen et al., 2017	Effects of pulmonary rehabilitation on exercise tolerance, quality of life, and emotional status in Parkinson's disease	Not power training
Azizi et al., 2014	Effects of water-based exercise on lower limb muscles strength in Parkinson's patients	Not power training
Myers et al., 2018	Effects of exercise on gait and motor imagery in people with Parkinson disease and freezing of gait	Not power training
Chiong et al., 2019	Effects of physical exercise on gait and balance in Parkinson's disease	Not power training
Maidan et al., 2018	Differential effects of two forms of exercise on prefrontal plasticity during walking in Parkinson's disease	Not power training

S3 Table. Excluded studies and reasons

Nakae et al., 2014	Effects of home exercise on physical function in Parkinson's disease patients	Not power training
Valverde-guijarro et al., 2022	Effects of contemporary dance and physiotherapy intervention on balance in Parkinson's disease	Not power training
Cheng et al., 2016	Effects of treadmill training on turning performance in Parkinson's disease	Not power training
Franzén et al., 2019	Effects of exercise on neuroplastic changes in Parkinson's disease	Not power training
Paolucci et al., 2014	Effects of perceptive rehabilitation on balance control in Parkinson's disease	Not power training
Chaves et al., 2011	Effects of physical therapy program in Parkinson disease	Not power training
Dereli et al., 2010	Effects of physiotherapist-supervised vs self-supervised exercise on quality of life in Parkinson's disease	Not power training
Gassner et al., 2022	Effects of individualized smartphone-based exercise program in Parkinson's disease	Not power training
De goede et al., 2004	Effects of group physiotherapy for Parkinson's disease	Not power training
Toole et al., 2000	Effects of balance and strength training on equilibrium in Parkinsonism	Not power training
Holmes et al., 2013	Effects of home-based VR rehabilitation on balance in Parkinson's disease	Not power training
Schilling et al., 2010	Effects of high-load resistance training on strength and function in Parkinson's disease	Not power training
Kanegusuku et al., 2021	Effects of resistance training on metabolic and cardiovascular response in Parkinson's disease	Not power training

S3 Table. Excluded studies and reasons

Cabrera-martos et al., 2020	Effects of core stabilization training on balance in Parkinson's disease	Not power training
Orcioli-silva et al., 2014	Effects of multimodal exercise on functional capacity in Parkinson's disease	Not power training
Shen et al., 2016	Effects of Exercise on Falls, Balance, and Gait Ability in Parkinson's Disease	Not power training
Gobbi et al., 2017	Effects of physical activity on walking in individuals with Parkinson's disease	Not power training
Son et al., 2018	The Effects of Mindfulness Meditation-Based Complex Exercise Program on Motor and Nonmotor Symptoms and Quality of Life in Patients with Parkinson's Disease	Not power training
Santos et al., 2016	Effects of leg muscle fatigue on gait in patients with Parkinson's disease and controls with high and low levels of daily physical activity	Not power training
Mohammadpour et al., 2018	Effects of a combined aerobic and resistance exercise program on the quality of life and motor function of elderly men with Parkinson's disease	Not power training
Allen et al., 2010	The effects of an exercise program on fall risk factors in people with Parkinson's disease: A randomized controlled trial	Not power training
Kuo et al., 2017	Effect of expiratory muscle strength training on maximum expiratory pressure and quality of life in PD	Not power training
Bazyar et al., 2022	Effect of Pilates and Aquatic Exercise on Muscle Strength and Range of Motion in PD	Not power training
Schilling et al., 2010	Effects of high-load lower-body resistance training on strength and function in PD	Not power training

S3 Table. Excluded studies and reasons

Toole et al., 2000	The effects of a balance and strength training program on equilibrium in Parkinsonism	Not power training
Azizi et al., 2014	Effects of water-based exercise on lower limb muscles strength in PD	Not power training
Carvalho et al., 2015	Comparison of strength, aerobic, and physical therapy for PD	Not power training
Krumpolec et al., 2017	Aerobic-strength exercise improves metabolism and clinical state in PD	Not power training
Clael et al., 2019	Strength and physical functions in people with PD	Not power training
Troche et al., 2014	Detraining outcomes with expiratory muscle strength training in PD	Not power training
Hüsch et al., 2022	Effect of strength training on orthostatic hypotension in PD	Not power training
Rossi et al., 2018	What makes a group fitness program for people with Parkinson's disease endure? A mixed-methods study of multiple stakeholders	Not power training
Ferrazzoli et al., 2018	Efficacy of intensive multidisciplinary rehabilitation in Parkinson's disease: A randomised controlled study	Not power training
Mantri et al., 2018	Physical activity in early Parkinson disease	Not power training
Sacheli et al., 2018	Habitual exercisers versus sedentary subjects with Parkinson's Disease: Multimodal PET and fMRI study	Not power training
De melo et al., 2018	Effect of virtual reality training on walking distance and physical fitness in individuals with Parkinson's disease	Not power training
Schroeteler et al., 2018	LSVT-BIG Training for people with Parkinson's disease	Not power training
Crowley et al., 2018	Exercise as therapy for Parkinson's?	Not power training

S3 Table. Excluded studies and reasons

Araújo et al., 2018	Physical exercise and evaluation of the functional capacity of an elderly with Parkinson	Not power training
Mulligan et al., 2018	Engagement in exercise for people with Parkinson's: What is meaningful?	Not power training
Giardini et al., 2018	Instrumental or physical-exercise rehabilitation of balance improves both balance and gait in Parkinson's disease	Not power training
Levy-tzedek et al., 2018	Improvement in upper-limb UPDRS motor scores following fast-paced arm exercise: A pilot study	Not power training
De carvalho et al., 2018	Physical exercise for Parkinson's disease: Clinical and experimental evidence	Not power training
Schroeteler et al., 2009	Has physical activity a disease modifying effect on Parkinson's disease?	Not power training
Nijkrake et al., 2009	Evaluation of a patient-specific index as an outcome measure for physiotherapy in Parkinson's disease.	Not power training
Ebersbach et al., 2014	Non-pharmacological treatment of Parkinson's disease.	Not power training
Tomlinson et al., 2014	Physiotherapy for parkinson's disease: A comparison of techniques.	Not original study
Fietzek et al., 2014	Physiotherapy leads to short term effects in PD patients with freezing of gait - A randomized crossover trial.	Not power training
O'callaghan et al., 2014	The effect of an exercise intervention on aerobic capacity in idiopathic Parkinson's disease.	Not power training
Mazilu et al., 2014	GaitAssist: A wearable assistant for gait training and rehabilitation in Parkinson's disease.	Not original study
Mak et al., 2021	Efficacy of a 6-week brisk walking program in improving non-motor	Not power training

S3 Table. Excluded studies and reasons

	symptoms in people with parkinson's disease-a randomized controlled trial.	
Law et al., 2023	Effects of an online 12-week Tai Chi intervention on the electromyography activity of the lower-limb muscles of individuals with Parkinson's disease during obstacle crossing.	Not power training
Linder et al., 2022	Increased comfortable gait speed is associated with improved gait biomechanics in persons with Parkinson's disease completing an 8-week aerobic cycling intervention	Not power training
Jansen et al., 2021	High intensity aerobic exercise improves bimanual coordination of grasping forces in Parkinson's disease	Not power training
Steib et al., 2018	A Single Bout of Aerobic Exercise Improves Motor Skill Consolidation in Parkinson's Disease	Not power training
Duchesne et al., 2015	Enhancing both motor and cognitive functioning in Parkinson's disease: Aerobic exercise as a rehabilitative intervention	Not power training
Silveira et al., 2018	Aerobic exercise is more effective than goal-based exercise for the treatment of cognition in Parkinson's disease	Not power training
Soto et al., 2023	Establishing the impact of aerobic exercise on biomarkers, mobility, and cognitive functioning of Parkinson's disease: A translational study	Not power training
Rosenfeldt et al., 2021	High intensity aerobic exercise improves information processing and motor performance in individuals with Parkinson's disease	Not power training

S3 Table. Excluded studies and reasons

Linder et al., 2022	An 8-week aerobic cycling intervention elicits improved gait velocity and biomechanics in persons with Parkinson's disease	Not power training
Marusiak et al., 2019	Eight weeks of aerobic interval training improves psychomotor function in patients with parkinson's disease—randomized controlled trial	Not power training
Skidmore et al., 2008	Pilot safety and feasibility study of treadmill aerobic exercise in Parkinson disease with gait impairment	Not original study
Levin et al., 2022	Aerobic training slows down the progression of motor symptoms of Parkinson's disease. Can an influence associated with this effect of aerobic training be proven by means of MRI in the networks involved in the disease process?	Not power training
Swank et al., 2016	The effect of aerobic exercise on dual-task gait in individuals with parkinson's disease	Not power training
Krumpolec et al., 2017	Aerobic-strength exercise improves metabolism and clinical state in Parkinson's disease patients	Not power training
Sacheli et al., 2012	The addition of aerobic or resistance training to sensory attention focused exercise: an enhanced treatment for Parkinson's disease?	Not power training
Di Martino et al., 2018	Aerobic rehabilitation program for improving muscle function in Parkinson's disease	Not power training
Bergen et al., 2002	Aerobic exercise intervention improves aerobic capacity and movement initiation in Parkinson's disease patients	Not power training

S3 Table. Excluded studies and reasons

Hortobágyi et al., 2022	The impact of aerobic and resistance training intensity on markers of neuroplasticity in health and disease	Not power training
Yaghoobi et al., 2019	Comparison of the effect of aerobic and resistance training on cardiovascular risk factors in Parkinson's women	Not power training
Koop et al., 2019	Mobility improves after high intensity aerobic exercise in individuals with Parkinson's disease	Not power training
Kishore et al., 2020	Effect of regular practice of yoga versus aerobic exercise on motor and non motor signs and motor cortex plasticity in Parkinson's disease	Not power training
Duchesne et al., 2016	Influence of aerobic exercise training on the neural correlates of motor learning in Parkinson's disease individuals	Not power training
Alberts et al., 2021	Effectiveness of a Long-Term, Home-Based Aerobic Exercise Intervention on Slowing the Progression of Parkinson Disease: Design of the Cyclical Lower Extremity Exercise for Parkinson Disease II (CYCLE-II) Study	Not power training
Penko et al., 2021	Effect of Aerobic Exercise on Cardiopulmonary Responses and Predictors of Change in Individuals With Parkinson's Disease	Not power training
Zhang et al., 2022	Effect of Zhan Zhuang Qigong on upper limb static tremor and aerobic exercise capacity in patients with mild-to-moderate Parkinson's disease: study protocol for a randomised controlled trial	Not power training
Soke et al., 2021	Task-oriented circuit training combined with	Not power training

S3 Table. Excluded studies and reasons

	aerobic training improves motor performance and balance in people with Parkinson's Disease	
Nadeau et al., 2018	A 12-Week Cycling Training Regimen Improves Upper Limb Functions in People With Parkinson's Disease	Not power training
Ridgel et al., 2012	Active-assisted cycling improves tremor and bradykinesia in Parkinson's disease	Not power training
Miner et al., 2020	Therapeutic effects of forced exercise cycling in individuals with Parkinson's disease	Not power training
Miner et al., 2023	Immediate effects of forced exercise cycling on core outcomes in individuals with Parkinson's disease	Not power training
Rosenfeldt et al., 2015	The cyclical lower extremity exercise for Parkinson's trial (CYCLE): Methodology for a randomized controlled trial	Not power training
Ridgel et al., 2009	Forced, not voluntary, exercise improves motor function in Parkinson's disease patients	Not power training
Hammond et al., 2017	Neuromuscular rate of force development deficit in Parkinson disease	Not power training
Smart et al., 2020	Importance of maximal strength and muscle-tendon mechanics for improving force steadiness in persons with parkinson's disease	Not power training
Corcos et al., 1996	Strength in Parkinson's disease: Relationship to rate of force generation and clinical status	Not power training
Ramsey et al., 2004	Muscle activation and force production in Parkinson's patients during sit to stand transfers	Not power training
Skinner et al., 2019	Lower Extremity Muscle Strength and Force Variability in Persons With Parkinson Disease	Not power training

S3 Table. Excluded studies and reasons

Kadkhodaie et al., 2020	Effect of eccentric-based rehabilitation on hand tremor intensity in Parkinson disease	Not power training
Mabry et al., 2023	The effect of nonimpact eccentric lower body exercise on Parkinson's symptoms and substantia nigra structure and function	Not power training
Santiago et al., 2023	Physical activity and lifestyle modifications in the treatment of neurodegenerative diseases	Not power training
Lee et al., 2023	Effects of proprioceptive neuromuscular facilitation on components of functional physical activity in patients with Parkinson's disease	Not power training
Cugusi et al., 2014	Effects of an adapted physical activity program on motor and non-motor functions and quality of life in patients with Parkinson's disease	Not power training
Sigurgeirsson et al., 2009	The effect of high-volume walking with visual cues on Gait in Parkinson's patients. A randomized controlled trial.	Not power training
Monteiro et al., 2017	Effects of Nordic walking training on functional parameters in Parkinson's disease: a randomized controlled clinical trial.	Not power training
Geroin et al., 2015	The effects of different gait training techniques for improving walking performance in patients in the early stages of Parkinson's Disease: a pilot randomized clinical trial.	Not power training
Cugusi et al., 2015	Effects of a Nordic Walking program on motor and non-motor symptoms, functional performance and body composition in patients with Parkinson's disease.	Not power training

S3 Table. Excluded studies and reasons

Beck et al., 2018	Can Dual Task Walking Improve in Parkinson's Disease After External Focus of Attention Exercise? A Single Blind Randomized Controlled Trial.	Not power training
Ambrus et al., 2019	Walking on a treadmill improves the stride length-cadence relationship in individuals with Parkinson's disease.	Not power training
Gougeon et al., 2017	Nordic Walking improves trunk stability and gait spatial-temporal characteristics in people with Parkinson disease.	Not power training
Dönmez et al., 2010	The effects of home exercise program on balance and functional capacity in parkinsonian patients.	Not power training
Nieuwboer et al., 2001	The effect of a home physiotherapy program for persons with Parkinson's disease.	Not power training
Nakae et al., 2011	Analysis of 24-h physical activities of patients with Parkinson's disease at home.	Not power training
Rodríguez et al., 2021	Home-Based Vigorous Tele-Exercise in People with Parkinson's Disease: Feasibility beyond Complexity.	Not original study
Nakae et al., 2014	Comparison of physical function and activity in home-care patients with Parkinson's disease and healthy elderly people.	Not power training
Esculier et al., 2012	Home-based balance training programme using Wii Fit with balance board for Parkinson's disease: A pilot study.	Not power training
Mak et al., 2020	Effect of home-based functional training program on enhancing gross and fine upper extremity function in people with Parkinson's	Not power training

S3 Table. Excluded studies and reasons

	disease-a randomized controlled trial.	
Jaywant et al., 2016	Randomized Controlled Trial of a Home-Based Action Observation Intervention to Improve Walking in Parkinson Disease.	Not power training
Manna et al., 2022	A Smart and Home-based Telerehabilitation Tool for Patients with Neuromuscular Disorder.	Not power training
Nuic et al., 2020	Serious games home rehabilitation program to treat gait and balance disorders in patients with Parkinson's disease: a randomised controlled trial.	Not power training
Slomski et al., 2018	High-Intensity Exercise Safe in De Novo Parkinson Disease.	Not power training
Szymura et al., 2020	Moderate-intensity balance training improve postural control in people with Parkinson's disease.	Not power training
Tollár et al., 2018	A High-Intensity Multicomponent Agility Intervention Improves Parkinson Patients' Clinical and Motor Symptoms.	Not power training
Cardalda et al., 2023	Is high intensity Pilates exercise treatment beneficial for people with Parkinson's disease?	Not power training
Handlery et al., 2024	High Intensity Functional Training for People with Parkinson's & Their Care Partners: A Feasibility Study.	Not original study
Cugusi et al., 2013	Body weight-supported high-intensity locomotor training and adapted physical activity program in patients with Parkinson's disease: A complementary approach?	Not power training
Mcneely et al., 2016	Quantity and intensity of physical activity in people with Parkinson's disease	Not power training

S3 Table. Excluded studies and reasons

	during exercise interventions.	
Kelly et al., 2014	Novel, high-intensity exercise prescription improves muscle mass, mitochondrial function, and physical capacity in individuals with Parkinson's disease.	Not power training
Zoladz et al., 2014	Moderate-intensity interval training increases serum brain-derived neurotrophic factor level and decreases inflammation in parkinson's disease patients.	Not power training
Walker et al., 2017	A randomised control trial to investigate if people with Parkinson's disease can exercise at high intensity, and does this improve their cardiorespiratory function? A feasibility study.	Not original study
Segura et al., 2020	Effect of a High-Intensity Tandem Bicycle Exercise Program on Clinical Severity, Functional Magnetic Resonance Imaging, and Plasma Biomarkers in Parkinson's Disease.	Not power training
Panassollo et al., 2024	Targeting exercise intensity and aerobic training to improve outcomes in Parkinson's disease.	Not power training
Swanton et al., 2018	Low-intensity, goal-directed occupational therapy and physiotherapy did not produce clinically meaningful differences in activities for people with mild to moderate Parkinson's Disease.	Not power training
Rose et al., 2013	Improved clinical status, quality of life, and walking capacity in parkinson's disease after body weight-supported high-intensity locomotor training.	Not power training

S3 Table. Excluded studies and reasons

Mcneely et al., 2016	Differential effects of tango, treadmill, and stretching interventions on gait in people with Parkinson's disease.	Not power training
Rabinovich et al., 2021	A high dose tango intervention for people with Parkinson's disease (PwPD).	Not power training
Stozek et al., 2016	Argentine tango in the rehabilitation of patients with Parkinson's disease.	Not power training
Hackney et al., 2007	Effects of tango on functional mobility in Parkinson's disease: A preliminary study.	Not power training
Blandy et al., 2015	Therapeutic Argentine tango dancing for people with mild Parkinson's disease: A feasibility study.	Not original study
Romenets et al., 2015	Tango for treatment of motor and non-motor manifestations in Parkinson's disease: A randomized control study.	Not power training
Albani et al., 2019	Feasibility of home exercises to enhance the benefits of tango dancing in people with Parkinson's disease.	Not original study
Seidler et al., 2016	Feasibility and preliminary efficacy of a telehealth approach to group tango instruction for people with Parkinson's disease.	Not original study
Sivertseva et al., 2022	Dance therapy in the rehabilitation of neurological diseases.	Not Parkinson's disease
Bognar et al., 2017	More than just dancing: experiences of people with Parkinson's disease in a therapeutic dance program.	Not power training
Haputhanthirige et al., 2023	Effects of dance on gait and dual-task gait in Parkinson's disease.	Not power training
De Natale et al., 2017	Dance therapy improves motor and cognitive functions in patients with Parkinson's disease.	Not power training

S3 Table. Excluded studies and reasons

Westbrook et al., 1989	Dance/movement therapy with groups of outpatients with Parkinson's disease.	Not power training
Hashimoto et al., 2015	Effects of dance on motor functions, cognitive functions, and mental symptoms of Parkinson's disease: A quasi-randomized pilot trial.	Not power training
Lee et al., 2018	Turo (Qi Dance) Program for Parkinson's Disease Patients: Randomized, Assessor Blind, Waiting-List Control, Partial Crossover Study.	Not power training
Natbony et al., 2013	Perceptions of a Videogame-Based Dance Exercise Program among Individuals with Parkinson's Disease.	Not power training
Lihala et al., 2021	Dance movement therapy in rehabilitation of Parkinson's disease – A feasibility study.	Not original study
Mckay et al., 2016	Balance, body motion, and muscle activity after high-volume short-term dance-based rehabilitation in persons with Parkinson disease: A pilot study.	Not power training
Marchant et al., 2010	Effects of a short duration, high dose contact improvisation dance workshop on Parkinson disease: A pilot study.	Not power training
Michels et al., 2018	"Dance Therapy" as a psychotherapeutic movement intervention in Parkinson's disease.	Not power training
Mazzarin et al., 2017	Effects of Dance and of Tai Chi on Functional Mobility, Balance, and Agility in Parkinson Disease.	Not power training
Westheimer et al., 2015	Dance for PD: a preliminary investigation of effects on motor function and quality of life among persons with Parkinson's disease (PD).	Not power training

S3 Table. Excluded studies and reasons

Rocha et al., 2018	Dance therapy for Parkinson's disease: A randomised feasibility trial. International Journal of Therapy and Rehabilitation	Not power training
Valverde-Guijarro et al., 2022	Effects of contemporary dance and physiotherapy intervention on balance and postural control in Parkinson's disease. Disability and Rehabilitation	Not power training
Stozek et al., 2013	Using dance in the rehabilitation of patients with Parkinson's disease. Rehabilitacja Medyczna	Not power training
Lee et al., 2015	Effect of virtual reality dance exercise on the balance, activities of daily living, and depressive disorder status of Parkinson's disease patients.	Not power training
Haas et al., 2023	Dance and Parkinson's: The effects on selected functional parameters during the 180 turning phase of the timed up & go test in people with Parkinson's. Journal of Parkinson's Disease	Not power training
Carapellotti et al., 2022	Evaluating the effects of dance on motor outcomes, non-motor outcomes, and quality of life in people living with Parkinson's: a feasibility study.	Not original study
Sidelkovskiy et al., 2022	Dance therapy in comprehensive rehabilitation of patients with Parkinson's disease.	Not power training
Allen et al., 2017	Increased neuromuscular consistency in gait and balance after partnered, dance-based rehabilitation in Parkinson's disease.	Not power training
Michels et al., 2018	A pilot study on dance/movement therapy in Parkinson's disease.	Not power training

S3 Table. Excluded studies and reasons

Moore et al., 2021	A Community-based Boxing Program is Associated with Improved Balance in Individuals with Parkinson's Disease.	Not power training
Sangarapillai et al., 2020	Are effects of Rock Steady Boxing and PD SAFExTM on the motor symptoms of Parkinson's disease maintained long-term?-A single-blinded randomized controlled trial.	Not power training
Watts et al., 2023	A Pilot Study of the Effect of a Non-Contact Boxing Exercise Intervention on Respiratory Pressure and Phonation Aerodynamics in People with Parkinson's Disease.	Not power training
Patel et al., 2023	A pilot study of a 12-week community-based boxing program for Parkinson's disease.	Not power training
Shearin et al., 2021	The effect of a multi-modal boxing exercise program on cognitive locomotor tasks and gait in persons with Parkinson disease.	Not power training
Domingos et al., 2019	Implementation of a Community-Based Exercise Program for Parkinson Patients: Using Boxing as an Example.	Not power training
Suarez-Gonzales et al., 2022	The application of a boxing training program in an individual with early-onset Parkinson's disease.	Not power training
Khuzema et al., 2020	Effect of home-based Tai Chi, Yoga or conventional balance exercise on functional balance and mobility among persons with idiopathic Parkinson's disease: An experimental study.	Not power training
Hall et al., 2011	Effect of a yoga programme on an individual with Parkinson's disease: A single-subject design.	Not power training

S3 Table. Excluded studies and reasons

Ujjan et al., 2022	Force Platform-Based Intervention Program for Individuals Suffering with Neurodegenerative Diseases like Parkinson.	Not power training
Howard et al., 2022	Measures of motor segmentation from rapid isometric force pulses are reliable and differentiate Parkinson's disease from age-related slowing.	Not power training
Beall et al., 2013	The effect of forced-exercise therapy for Parkinson's disease on motor cortex functional connectivity.	Not power training
Lin et al., 2022	Effects of Lower Limb Cycling Training on Different Components of Force and Fatigue in Individuals With Parkinson's Disease.	Not power training
Auff et al., 1995	Parkinson's disease and neurological rehabilitation.	Not original study
Nascimento et al., 2014	Effect of a multimodal exercise program on sleep disturbances and instrumental activities of daily living performance on Parkinson's and Alzheimer's disease patients.	Not power training
Canning et al., 2008	Multiple-task walking training in people with mild to moderate Parkinson's disease: A pilot study.	Not power training
Rochester et al., 2015	Multidisciplinary rehabilitation in Parkinson's disease: A milestone with future challenges.	Not power training
Ashburn et al., 2018	The PDSAFE falls prevention programe for people with Parkinson's: a multicentre randomised controlled trial.	Not power training
Siega et al., 2022	Multicomponent Aquatic Training (MAT) Program for People with Parkinson's Disease: A Protocol for a Controlled Study.	Not power training

S3 Table. Excluded studies and reasons

Ferrusola-Pastrana et al., 2023	The therapeutic effects of multimodal exercise for people with Parkinson's: A longitudinal community-based study.	Not power training
Mafra et al., 2022	Multimodal exercise program contributes to balance and motor functions in men and women with Parkinson's disease differently: an intervention study.	Not power training
Penko et al., 2019	Multimodal training reduces fall frequency as physical activity increases in individuals with Parkinson's disease.	Not power training
Munneke et al., 2012	Efficacy of a multifaceted intervention program to increase physical activity in patients with PD: the parkfit trial.	Not power training
Raccagni et al., 2019	Physiotherapy improves motor function in patients with the Parkinson variant of multiple system atrophy: A prospective trial.	Not power training
Monticone et al., 2015	In-patient multidisciplinary rehabilitation for Parkinson's disease: A randomized controlled trial.	Not power training
Goodwin et al., 2015	A multi-centre, randomised controlled trial of the effectiveness of PDSAFE to prevent falls among people with Parkinson's: Study protocol.	Not power training
Carne et al., 2005	Efficacy of multidisciplinary treatment program on long-term outcomes of individuals with Parkinson's disease.	Not power training
Brittle et al., 2008	Short-term effects on mobility, activities of daily living and health-related quality of life of a Conductive Education programme for adults with multiple sclerosis,	Not power training

S3 Table. Excluded studies and reasons

	Parkinson’s disease and stroke.	
Hulbert et al., 2021	‘PDSAFE’ - a multi-dimensional model of falls-rehabilitation for people with Parkinson’s. A mixed methods analysis of therapists’ delivery and experience.	Not power training
Rosenfeldt et al., 2019	Improvements in temporal and postural aspects of gait vary following single- and multi-modal training in individuals with Parkinson’s disease.	Not power training
Chivers et al., 2019	Multicentre, randomised controlled trial of PDSAFE, a physiotherapist-delivered fall prevention programme for people with Parkinson’s.	Not power training
Speelman et al., 2014	Evaluation of implementation of the ParkFit program: A multifaceted intervention aimed to promote physical activity in patients with Parkinson’s disease.	Not power training
La Porta et al., 2022	Efficacy of a multiple-component and multifactorial personalized fall prevention program in a mixed population of community-dwelling older adults with stroke, Parkinson’s Disease, or frailty compared to usual care: The PRE.C.I.S.A. randomized controlled trial.	Not power training
De Faria et al., 2023	Multicomponent and mat Pilates training increased gait speed in individuals with Parkinson’s disease when walking and carrying a load: A single-blinded randomized controlled trial.	Not power training
Saluja et al., 2023	Multi-modal rehabilitation therapy in Parkinson’s disease and related disorders.	Not power training

S3 Table. Excluded studies and reasons

Pelosin et al., 2020	A multimodal training modulates short afferent inhibition and improves complex walking in a cohort of faller older adults with an increased prevalence of Parkinson's disease.	Not power training
Frazzitta et al., 2014	Multidisciplinary intensive rehabilitation treatment and rotigotine in the early stages of Parkinson's disease: a randomized controlled study.	Not power training
Goffredo et al., 2023	Efficacy of non-immersive virtual reality-based telerehabilitation on postural stability in Parkinson's disease: a multicenter randomized controlled trial.	Not power training
Cohen et al., 2021	Multidisciplinary intensive outpatient rehabilitation program for patients with moderate-to-advanced Parkinson's disease.	Not power training
Mestre et al., 2009	A multimodal approach to physical therapy in Parkinson's disease: Optimizing strategies.	Not power training
Seymour et al., 2018	'PDSAFE'-A multi-dimensional fall rehabilitation intervention for people with Parkinson's with specialist physiotherapy training: qualitative exploration of physio's experiences.	Not power training
Sedaghati et al., 2016	A selective corrective exercise to decrease falling and improve functional balance in idiopathic Parkinson's Disease.	Not power training
Duncan et al., 2014	Are the effects of community-based dance on Parkinson disease severity, balance, and functional mobility reduced with time? A 2-year prospective pilot study.	Not power training

S3 Table. Excluded studies and reasons

Cerchiario et al., 1993	Functional rehabilitation in Parkinson disease.	Not power training
Ke et al., 2022	Effects of 12 weeks of Tai Chi Chuan intervention on the postural stability and self-reported instability in subjects with functional ankle instability: Study protocol for a randomized controlled trial.	Not power training
Schilling et al., 2009	Impaired leg extensor strength in individuals with Parkinson disease and relatedness to functional mobility.	Not power training
Cubo et al., 2021	Adopting a multidisciplinary telemedicine intervention for fall prevention in Parkinson's disease. Protocol for a longitudinal, randomized clinical trial.	Not power training
Ghielen et al., 2015	BEWARE: Body awareness training in the treatment of wearing-off related anxiety in patients with Parkinson's disease: Study protocol for a randomized controlled trial.	Not power training
Law et al., 2023	Effects of a biomechanical-based Tai Chi program on gait and posture in people with Parkinson's disease: study protocol for a randomized controlled trial.	Not original study
Loidl et al., 2023	Implementation and the effects of a Parkinson Network Therapy (PaNTher) on activities of daily living and health-related quality of life in Parkinson's disease patients: Study protocol of an mixed-method observational cohort study in outpatient care.	Not power training
Magaña et al., 2023	Investigating the impact of physical activity on mitochondrial function in Parkinson's disease	Not power training

S3 Table. Excluded studies and reasons

	(PARKEX): Study protocol for a randomized controlled clinical trial.	
Patterson et al., 2022	Study in Parkinson's disease of exercise phase 3 (SPARX3): study protocol for a randomized controlled trial.	Not power training
Cheung et al., 2019	The protocol for a combined upper limb exercise and Do-It- Yourself community program for people with Parkinson's disease.	Not power training
Zoetewei et al., 2021	Protocol for the DeFOG trial: A randomized Parkinson's disease.	Not power training
Harpham et al., 2024	Home-based high-intensity interval training for people with Parkinson's: Protocol for the HIIT-Home4Parkinson's randomized, controlled feasibility study.	Not original study
Nascimento et al., 2019	Effects of motor imagery training of Parkinson's disease: A protocol for a randomized clinical trial.	Not power training
Leavy et al., 2017	Evaluation and implementation of highly challenging balance training in clinical practice for people with Parkinson's disease: Protocol for the HiBalance effectiveness-implementation trial.	Not power training
Zelada-astudillo et al., 2021	Effect of the combination of automated peripheral mechanical stimulation and physical exercise on aerobic functional capacity and cardiac autonomic control in patients with Parkinson's disease: a randomized clinical trial protocol.	Not power training
Kashif et al., 2020	Effects of Virtual Reality with Motor Imagery Techniques in Patients with Parkinson's Disease: study	Not power training

S3 Table. Excluded studies and reasons

	Protocol for a Randomized Controlled Trial.	
Canning et al., 2009	Exercise therapy for prevention of falls in people with Parkinson's disease: A protocol for a randomised controlled trial and economic evaluation.	Not power training
Moratelli et al., 2022	Functional training versus Mat Pilates in motor and non-motor symptoms of individuals with Parkinson's disease: study protocol for a randomized controlled trial.	Not power training
Advocat et al., 2013	The effects of a mindfulness-based lifestyle programme for adults with Parkinson's disease: Protocol for a mixed methods, randomised two-group control study.	Not power training
Frazzitta et al., 2009	Rehabilitation treatment of gait in patients with Parkinson's disease with freezing: A comparison between two physical therapy protocols using visual and auditory cues with or without treadmill training.	Not power training
Morris et al., 2011	Falls and mobility in Parkinson's disease: Protocol for a randomised controlled clinical trial.	Not power training
Strouwen et al., 2014	Protocol for a randomized comparison of integrated versus consecutive dual task practice in Parkinson's disease: The DUALITY trial.	Not power training
Annesi et al., 2019	Effects of a Group Protocol on Physical Activity and Associated Changes in Mood and Health Locus of Control in Adults with Parkinson Disease and Reduced Mobility.	Not power training
Campani et al., 2022	The prevention of falls in patients with Parkinson's	Not power training

S3 Table. Excluded studies and reasons

	disease with in-home monitoring using a wearable system: a pilot study protocol.	
Brauer et al., 2011	Single and dual task gait training in people with Parkinson's Disease: A protocol for a randomised controlled trial.	Not power training
Arroyo-ferrer et al., 2021	Validation of cognitive rehabilitation as a balance rehabilitation strategy in patients with Parkinson's disease: Study protocol for a randomized controlled trial.	Not power training
Coe et al., 2018	Physical Activity, Fatigue, and Sleep in People with Parkinson's Disease: A Secondary per Protocol Analysis from an Intervention Trial.	Not power training
Gong et al., 2022	Commentary: A Longitudinal Randomized Controlled Trial Protocol to Evaluate the Effects of Wuqinxi on Dynamic Functional Connectivity in Parkinson's Disease Patients.	Not power training
Magana et al., 2023	Investigating the impact of physical activity on mitochondrial function in Parkinson's disease (PARKEX): study protocol for A randomized controlled clinical trial.	Not power training
Mavrommati et al., 2017	Exercise response in Parkinson's disease: Insights from a cross-sectional comparison with sedentary controls and a per-protocol analysis of a randomised controlled trial.	Not power training
Behrman et al., 2000	Practice as an intervention to improve speeded motor performance and motor learning in Parkinson's disease.	Not power training

S3 Table. Excluded studies and reasons

Welman et al., 2018	Therapist-supervised compared to home-based balance training encourages a “posture first” strategy during turn-to-sit transitions in individuals with Parkinson’s disease.	Not power training
Atterbury et al., 2016	Balance training in individual’ns with Parkinson’s disease: therapist-supervised vs. home-based exercise programmes.	Not power training
Zhou et al., 2018	Nordic walking improves gait power profiles at the knee joint in Parkinson’s disease.	Not power training
Stiles et al., 2020	Motor Performance and Quality of Life in a Community Exercise Program for Parkinson Disease.	Not power training
Van Nimwegen et al., 2010	Design and baseline characteristics of the ParkFit study, a randomized controlled trial evaluating the effectiveness of a multifaceted behavioral program to increase physical activity in Parkinson patients.	Not power training
Cohen et al., 2023	Multidisciplinary Intensive Rehabilitation Program for People with Parkinson’s Disease: Gaps between the Clinic and Real-World Mobility.	Not power training
Zawar et al., 2023	PowerUp: A Comprehensive virtual program for managing pain in Parkinson’s disease.	Not power training
King et al., 2020	Cognitively Challenging Agility Boot Camp Program for Freezing of Gait in Parkinson Disease.	Not power training
Debû et al., 2023	Impact of active gait training programs on gait under daily life conditions	Not power training

S3 Table. Excluded studies and reasons

	and quality of life in Parkinson's disease.	
Miletić et al., 2014	The effect of exercise programs on fall prevention for people with Parkinson's disease.	Not power training
Lavin et al., 2020	Rehabilitative Impact of Exercise Training on Human Skeletal Muscle Transcriptional Programs in Parkinson's Disease.	Not power training
Lee et al., 2015	Effects of virtual reality exercise program on balance and quality of life among patients with Parkinson's disease.	Not power training
Lai et al., 2020	Exploring the uptake and implementation of tele-monitored home-exercise programmes in adults with Parkinson's disease: A mixed-methods pilot study.	Not power training
King et al., 2017	A combined cognitive and motor exercise program for people with Parkinson's disease and Freezing of gait; a pilot study.	Not power training
Ward et al., 2000	Changes in maximum capacity tongue function following the Lee Silverman Voice Treatment Program.	Not power training
Hubble et al., 2016	PERFORM: rationale and design for a controlled study in fluctuating PD patients examining the effects of motor state on the outcomes from a structured physical therapy (PT) program.	Not power training
Gruber et al., 2008	Self-management programs for people with Parkinson's disease: A program evaluation approach.	Not power training
Harpham et al., 2023	Co-Creating a Feasible, Acceptable and Safe Home-Based High-Intensity Interval Training Programme for People with	Not power training

S3 Table. Excluded studies and reasons

	Parkinson's: The HIIT-Home4Parkinson's Study.	
Cleary et al., 2020	Parkinson's Disease: Exploring Motives for Long-Term Adherence to a Group Exercise Program.	Not power training
Ayán et al., 2014	Effects of two different exercise programs on gait parameters in individuals with Parkinson's disease: A pilot study.	Not power training
Waddell et al., 2021	The Feasibility of a Remote Physical Activity Monitoring Program for Rural Veterans with Stroke or Parkinson's Disease.	Not power training
Carlson-Kuhta et al., 2019	Effects of cognitively challenging agility exercise program on clinical and objective measures in people with Parkinson's Disease.	Not power training
Pérez-De La Cruz et al., 2016	Effects of an Ai Chi fall prevention programme for patients with Parkinson's disease.	Not power training
Hubble et al., 2016	PERFORM: controlled study in fluctuating PD patients examining the effects of motor state on the outcomes resulting from a structured physical therapy (PT) program.	Not power training
Wagner et al., 2022	Evaluation of an individualized, tablet-based physiotherapy training programme for patients with Parkinson's disease: the ParkProTrain study, a quasi-randomised controlled trial.	Not power training
Fernández et al., 2008	Physical therapy program improves motor skills in the long term in patients with Parkinson's disease.	Not power training
King et al., 2012	Comparison of individual, group and home agility boot camp (ABC) exercise	Not power training

S3 Table. Excluded studies and reasons

	program for people with Parkinson's disease.	
Domingos et al., 2021	A novel boot camp program to help guide personalized exercise in people with Parkinson disease.	Not power training
Tollár et al., 2019	Vastly Different Exercise Programs Similarly Improve Parkinsonian Symptoms: a Randomized Clinical Trial.	Not power training
Collett et al., 2017	Phase II randomised controlled trial of a 6-month self-managed community exercise programme for people with Parkinson's disease.	Not power training
Paul et al., 2021	People with Parkinson's disease are more willing to do additional exercise if the exercise program has specific attributes: a discrete choice experiment.	Not power training
Ploughman et al., 2014	Effectiveness of a novel community exercise transition program for people with moderate to severe neurological disabilities.	Not power training
Khobkhun et al., 2021	Can a targeted home-based exercise programme improve turning characteristics in individuals with Parkinson's disease?	Not power training
Delabays et al., 2001	Parkinson's disease: About an interdisciplinary rehabilitation program.	Not power training
