# Supplementary material for: Effect of power training on physical functional performance of patients with Parkinson’s disease: A systematic review and meta-analysis of randomized controlled trials
Source: PLoS One. 2025 Feb 4;20(2):e0314058. doi: 10.1371/journal.pone.0314058 (PMC11793793; doi:10.1371/journal.pone.0314058)
Supplement: Attachment — Submitted filename: Letter to reviewers.docx [file pone.0314058.s004.docx]

PONE-D-23-42955

**EFFECT OF POWER TRAINING ON PHYSICAL FUNCTIONAL PERFORMANCE OF PATIENTS WITH PARKINSON’S DISEASE: A SYSTEMATIC REVIEW AND META-ANALYSIS OF RANDOMIZED CONTROLLED TRIALS PLOS ONE**

Dear Editors and Reviewers,

We thank you for your comments and the time you spent appraising our manuscript. Below you may find a point-by-point answer to all of your questions and requirements. Editorial requests and reviewers' comments are written in *italic* and amendments we have made are in red in this file and highlighted in the main manuscript. We removed the funding statement from the acknowledgment. Actually, we didn't have financial support for this manuscript.

Yours sincerely,

Pedro Braga Neto

*Journal Requirements:*

*When submitting your revision, we need you to address these additional requirements.*

*1. Please ensure that your manuscript meets PLOS ONE's style requirements, including those for file naming. The PLOS ONE style templates can be found at*

*https://journals.plos.org/plosone/s/file?id=wjVg/PLOSOne_formatting_sample_main_body.pdf and*

[*https://journals.plos.org/plosone/s/file?id=ba62/PLOSOne_formatting_sample_title_authors_affiliations.pdf*](https://journals.plos.org/plosone/s/file?id=ba62/PLOSOne_formatting_sample_title_authors_affiliations.pdf)

Response: Thank you for the comment. We have formated the manuscript according to the journal’s style.

*2. Thank you for stating the following in the Acknowledgments Section of your manuscript:*

*"The author Pedro Braga Neto received funding from the Brazilian National Council for Scientific and Technological Development (CNPq) as research grant funding (Productivity scholarship). The author Samuel Brito de Almeida received funding from Ceará Foundation of Support to Scientific and Technological Development (FUNCAP) as doctoral scholarship. The author Emamnuelle Silva Tavares Sobreira received a research grant funding (Scientific and regional development scholarship) from CNPQ/FUNCAP."*

*We note that you have provided funding information that is not currently declared in your Funding Statement. However, funding information should not appear in the Acknowledgments section or other areas of your manuscript. We will only publish funding information present in the Funding Statement section of the online submission form.*

*Please remove any funding-related text from the manuscript and let us know how you would like to update your Funding Statement. Currently, your Funding Statement reads as follows:*

*"The author(s) received no specific funding for this work."*

*Please include your amended statements within your cover letter; we will change the online submission form on your behalf.*

Response: We apologize for this mistake. We removed the funding information at the acknowledgment section. Indeed, this funding information was not related to this work. It is a scholarship grant from the corresponding author. So, we removed it from the system.

*3. Please upload a new copy of Figures 1 to 7 as the detail is not clear. Please follow the link for more information:*

*https://blogs.plos.org/plos/2019/06/looking-good-tips-for-creating-your-plos-figures-graphics/*

*https://blogs.plos.org/plos/2019/06/looking-good-tips-for-creating-your-plos-figures-graphics/*

Response: Thank you for this suggestion. After consulting the links sent, we changed the figures, seeking to improve them. The new version of the figures has 700dpi of resolution and are saved as TIFF.

*4. Please include captions for your Supporting Information files at the end of your manuscript, and update any in-text citations to match accordingly. Please see our Supporting Information guidelines for more information:* [*http://journals.plos.org/plosone/s/supporting-information*](http://journals.plos.org/plosone/s/supporting-information)*.*

Response: We apologize for this mistake. We now included captions at the end of the manuscript.

*5. We note that this manuscript is a systematic review or meta-analysis; our author guidelines therefore require that you use PRISMA guidance to help improve reporting quality of this type of study. Please upload copies of the completed PRISMA checklist separately as Supporting Information with a file name “PRISMA checklist".*

Response: We thank you for this comment. We now included the PRISMA checklist file.

*Additional Editor Comments:*

*Interesting study; would suggest*

*1- Define your study PICO precisely;*

Response: We thank the editor for the comments. The PICO strategy is precisely defined on page 6, lines 112 to 138, as you can see below:

The inclusion criteria were defined based on the participants involved, type of intervention, type of comparison group, outcome of interest and study design (PICOS strategy) (19). Therefore, the following inclusion criteria were considered: (P) participants with confirmed PD diagnosis fulfilling the British Brain Bank Criteria or Movement Disorders Society criteria at any age or disease stage and community-dwelling people (20); (I) interventions: training program that included power training as a single strategy (machines, free weights, elastic tubing, weighted vests, elastic bands). All power training program, regardless of intensity, frequency, or session duration were considered eligible; (C): For the comparator group, we included studies in which participants had no intervention, usual care for the given study setting, wait-list trials incorporating a placebo-based PA program (sham exercises), another form of PA intervention, or drug therapy. Examples include but are not limited to: balance training, training of any type of strength manifestation (resistance training, among others), aerobic exercise, Tai Chi, movement strategy training, fall prevention education and home exercise prescription. We excluded trials that compared PT versus nutritional supplementation; (O): PFP was considered as the primary outcome. For this, we considered the following tests: 1) mobility tests (Five Times Sit-To-Stand, 30-sec chair stand, Backward walking, Timed Up and Go (TUG), Timed Up and Go dual task and the Short Physical Performance Battery); 2) gait tests (fast and comfortable 10 Meter Walk, 4-Meter Walk, preferred walking speed (PWS) and fast walking speed (FWS), Dynamic Gait Index, Modified Gait Efficacy Scale and 6-min walk); and 3) balance tests (Berg Balance Scale (BBS), single leg stance (SLS), Fullerton Advanced Balance scale, Mini-BESTest, Performance-Oriented Mobility Assessment, Biodex Balance and Activities Specific Balance Confidence Scale). Data regarding these outcomes should be presented at pre and post moments or the change values between these moments. When necessary, the authors were contacted for data recovery; finally, (S): The following study designs were considered: randomized controlled trials (RCTs) and quasi-randomized controlled trials.

*2- Please be sure about the literature review and comprehensive search in all relevant debases;*

Response: We thank the editor for this recommendation. Aiming to improve the literature search, we conducted additional searches in Cochrane Library and Scopus databases in April, 2024. The search strategy adopted is available in the Supporting Information (S2 File).

*3- Please employ ROB II in order to evaluate risk of bias as you see in* [*https://methods.cochrane.org/risk-bias-2*](https://methods.cochrane.org/risk-bias-2)

Response: We thank the editor for this recommendation. We now included the ROB II to evaluate the risk of bias. The information is added in the methods section (pages 8 and 9, lines 184 to 202) and in the results section (pages 23 and 24, lines 367 to 386) and figure 7.

The methodological quality of the included trials was evaluated using the Revised Cochrane risk-of-bias tool for randomized trials (RoB 2) [23]. This tool assesses bias across five key domains: 1) bias arising from the randomization process, 2) bias due to deviations from intended interventions, considering both the effect of assignment to the intervention and adherence to it, 3) bias due to missing outcome data, 4) bias in the measurement of the outcome, and 5) bias in the selection of the reported results. Each domain is examined through 3–7 signaling questions, with possible responses including: yes, probably yes, probably no, no, and no information. Based on the responses to these questions, an algorithm determines the risk of bias for each domain, categorizing it as “low risk of bias,” “high risk of bias,” or “some concerns.”

After evaluating each domain individually, an overall risk of bias judgment for each study was made using the same classification criteria. A study was deemed to have a “low risk of bias” if all domains met the required standards. If at least one domain raised “some concerns” or lacked clarity, the study was classified under “some concerns.” A “high risk of bias” designation was assigned when at least one domain did not meet the standards, or when multiple domains were classified as having “some concerns.” Two independent reviewers (SB and DP) assessed the risk of bias of the included studies, and any disagreements in the scoring of trials were resolved with a third reviewer.

The summary of the risk of bias analysis is depicted in Fig 7, according to the RoB2 tool [24]. Overall, the studies exhibited varying levels of risk. Two studies [12,14] demonstrated a high risk of bias across multiple domains, particularly in domains related to randomization and measurement of the outcome (D1 and D3). One study [15] showed some concerns, primarily in domain 5, related to the appropriateness of the analysis considering the missing data and deviations from the intended intervention. Another study [13] displayed a mix of low risk in randomization but concerns or high risks in other areas, especially in domain 4, which relates to the influence of missing data on the outcomes.

The high risk in domain 1 for some studies [12,15] was attributed to the lack of clarity on the randomization process. Similarly, domain 3 had concerns due to potential deviations from the intended interventions that were not accounted for in the analysis [12,14]. Domain 5, concerning selective reporting of outcomes, also contributed to the overall concerns as it was unclear whether the outcomes were analyzed as per the pre-specified plans [13]. These issues collectively led to an overall categorization of high risk for some studies and some concerns for others.

*Fig. 7 Summary of risk of bias by domain and study.*

*4- Apparently seems the results of this systematic review has high heterogeneity. Heterogeneity is an important consideration in systematic reviews, as high heterogeneity may imply that it is not suitable to perform meta-analysis.*

Response: We thank you for your feedback regarding the observed heterogeneity in the results of our systematic review. We appreciate your concern and acknowledge the importance of addressing heterogeneity in systematic reviews. We have developed a text that takes into account the issue of heterogeneity of results; however, we argue the relevance of our meta-analysis. We included the text in the limitations (page 28 and 29, lines 491 to 510), as follows.

Among the six meta-analyses conducted, high heterogeneity was indeed observed in two (related to PWS and FWS outcomes). In the remaining four meta-analyses, heterogeneity was 0%. We understand that high heterogeneity represents an opportunity to explore potential differences among the included studies. In this context, we sought to assess the feasibility of conducting sensitivity analyses and meta-regressions. However, in attempting to meet the prerequisites for such analyses, we found that it could not be performed. According to the Cochrane Handbook, meta-regressions are viable only when there are at least 10 studies/comparisons for each outcome. In both analyses (where heterogeneity was high), we had only 2 studies included, which precludes the possibility of performing sensitivity analyses and meta-regressions due to limited literature on the topic. Further, in an effort to understand the heterogeneity, we qualitatively analyzed the two included studies, particularly regarding their samples and methodologies. Upon careful review, we observed that the study by Meng et al. (2015) included an older sample with less severe disease symptoms (UPDRS-III) compared to the study by Paul et al. (2013). Additionally, the training protocols differ somewhat (Meng's protocol included more exercises and greater intensity compared to Paul's). We believe these differences between the studies may have led to the varying adaptations in PWS and FWS observed, resulting in high heterogeneity. However, it is important to note that both studies meet the eligibility criteria for this review and adhere to the established PICOT criteria.

Additionally, we have utilized random-effects models and applied appropriate statistical techniques to account for heterogeneity when pooling data.

We acknowledge that heterogeneity exists within the body of evidence synthesized in our review, but we would like to keep these 2 meta-analyses in our paper, considering it can still provide valuable insights by quantitatively summarizing the available data and identifying trends across studies.

Ultimately, we believe that our systematic review, despite the observed heterogeneity, contributes valuable information to the field and can serve as a basis for further research and clinical decision-making. We are committed to transparently reporting our methods and findings to ensure the integrity and reliability of our research.

***5. Review Comments to the Author***

***Reviewer #1:*** *Thank you for submitting your manuscript for consideration. Your topic choice is an excellent opportunity for a systematic review. However, I have identified several areas where improvements are necessary to meet the publication standards:*

*Abstract Structure: The abstract currently lacks an introduction section. I also noticed that certain abbreviations, such as "PFP," are used in the abstract without prior definition.*

Response: Thank you for these recommendations. In the revised version of the manuscript, we added two sentences of introduction in the abstract (page 2) and we defined the abbreviations presented.

*Literature Search: The literature search appears to be limited in scope. For a comprehensive review, it is essential to include major databases such as COCHRANE and Scopus. for example, wondering why this important study is not included? https://pubmed.ncbi.nlm.nih.gov/33927114/*

Response: We thank the reviewer for this recommendation. Aiming to improve the literature search, we conducted searches in Cochrane Library and Scopus in April, 2024. The complete search strategy adopted is available in the Supporting Information (S2 Table).

Regarding the study you recommend, it's not eligible considering the intervention proposed. As previously registered in our protocol (Prospero registration CRD42019139446) to be eligible, the interventions should be a training program that includes power training as a single strategy. So, the study of Strand et al. (2021) was excluded considering this reason.

*Presentation of Findings: The major findings of the review are not summarized in Table 1. Consider creating an additional table (table 2) that shows the effects of the intervention in each study. This table should ideally compare the differences in outcome measures from baseline, which will enhance the clarity and impact of your results.*

Response: We thank the reviewer for the valuable suggestion regarding the presentation of findings in our systematic review. In response to your recommendation, we have created an additional table (Table 2) that summarizes the effects of the intervention in each study. This new table compares the differences in outcome measures from baseline, providing a clearer and more impactful presentation of our results. We believe that this addition enhances the overall clarity of the findings and better supports the conclusions drawn from our analysis. We appreciate your guidance in improving the quality of our manuscript.

*Formatting: The references are not formatted according to the PLOS ONE style guide.*

*For references, we use the topic "online articles" as a source. This information was based on the submission guidelines (topic Formatting references). However, we can change to the topic " Published articles”.*

Response: We apologize for this mistake. The references are now formatted according to the PLOS ONE style guide.

*Figures: Titles are missing for the plots and figures, and their captions are incorrectly placed in the manuscript texts instead of below the figures. Additionally, to enhance the readability and accessibility of your table, I recommend numbering each abbreviation mentioned within the tables and providing a keyed list of these abbreviations below the table.*

Response: We appreciate this timely correction. We have changed the positioning of the figure captions to immediately below each one. Regarding the tables, we chose to keep the abbreviations explained in footnotes, without numbering, because we believe that this way it is cleaner and clearer for readers. We have seen that recent publications in PLOS ONE adopt this same standard for abbreviations in tables. For example:

Dong J, Wang D, Zhong S. Effects of different exercise types and cycles on pain and quality of life in breast cancer patients: A systematic review and network meta-analysis. PLoS One. 2024 Jul 3;19(7):e0300176. doi: 10.1371/journal.pone.0300176. PMID: 38959209; PMCID: PMC11221662.

*Reviewer #2: Introduction*

*The article cited to support the phrase "Additionally, muscle power is considered a better predictor of muscle function than strength (7)" refers to a review whose findings do not provide consistent evidence to support this statement.*

Response: We thank the reviewer for bringing this concern to our attention regarding the citation used to support the statement "Additionally, muscle power is considered a better predictor of muscle function than strength." We appreciate your diligence in scrutinizing the references provided in our manuscript. Upon reevaluation, we have identified another robust reference that more accurately supports the aforementioned statement. We apologize for any confusion caused by the initial citation and assure you that we have replaced it with a more appropriate and relevant source. We understand the importance of ensuring the accuracy and validity of the evidence cited in our manuscript and have taken steps to address this issue promptly. The new reference provides stronger and more consistent evidence to support the assertion regarding muscle power as a predictor of muscle function. We sincerely appreciate your feedback and the opportunity to improve the quality of our manuscript.

Reference: Freitas, S.R., Cruz-Montecinos, C., Ratel, S. et al. Powerpenia Should be Considered a Biomarker of Healthy Aging. Sports Med - Open 10, 27 (2024). https://doi.org/10.1186/s40798-024-00689-6

*It is essential for the authors to clearly state the gap in the field of knowledge that the manuscript will address. The studies cited to suggest controversies in the literature (12, 14, and 15) utilized different types of exercises and equipment; therefore, they do not necessarily present opposing results but rather different responses to varying exercise doses.*

Response: Thank you for your valuable feedback. Based on your suggestion, we have revised the introduction to clearly define the gap in the literature and address the variability in exercise protocols across studies. Specifically, we added the following (page 4, lines 77 to 83 and 86 to 90):

"In reviewing the literature, we identified a range of exercise protocols under the umbrella of PT, including variations in equipment, intensity, and duration, leading to seemingly contradictory findings. For example, while some studies reported improvements in gait velocity, balance, and mobility with PT (12,13), others did not observe significant gains in these same outcomes (14,15). These discrepancies are not merely conflicting results but rather reflect the variability in exercise prescription across studies, which complicates the interpretation of PT’s effectiveness in this population.

The primary gap in the current literature is the lack of a clear consensus on the optimal PT protocol for improving PFP in PD patients, considering the diversity in exercise modalities and dosing. Our systematic review and meta-analysis aim to address this gap by synthesizing evidence from randomized controlled trials (RCTs) to clarify the overall impact of PT on PFP in PD patients, while accounting for variations in exercise interventions."

*It is imperative to clarify in the introduction the meaning of "physical functional performance" as the set of tests indicated in the method to assess the primary outcome (mobility tests such as Five Times Sit-To-Stand, 30-sec chair stand, Backward walking, Timed Up and Go (TUG), Timed Up, and Go dual-task, and the Short Physical Performance Battery) measure distinct parameters. The study's objective is clearly defined.*

Response: We thank the reviewer for your continued insightful feedback. We appreciate your suggestion to clarify the term "physical functional performance" (PFP) within the context of our study. Based on your comments, we have revised the introduction (pages 3 and 4, lines 62 to 72) to more explicitly define PFP and its relevance to the tests used in our methodology.

Physical functional performance (PFP) refers to an individual's ability to perform tasks that require physical movement and strength, which are essential for independent living and quality of life, particularly in populations with functional impairments such as Parkinson's Disease (PD). PFP encompasses various domains, including strength, balance, mobility, and endurance, each of which contributes to an individual's overall functional capacity. In our study, PFP is specifically operationalized through a series of standardized mobility tests that are widely recognized in the literature for assessing key aspects of physical function in PD patients, including the Five Times Sit-To-Stand (FTSTS), 30-Second Chair Stand, Backward Walking, Timed Up and Go (TUG), TUG Dual-Task, and the Short Physical Performance Battery (SPPB). These tests collectively provide a multifaceted evaluation of PFP, capturing the diverse aspects of physical function that are often compromised in PD patients.

*Method*

*Participants: In the inclusion criteria, why did the authors include studies with Parkinson's patients in different stages of the disease? Is the response to an exercise-based intervention not influenced by the disease stage?*

Response: We thank the reviewer for this pertinent question regarding the inclusion of studies involving Parkinson’s Disease (PD) patients at different stages. We appreciate the opportunity to clarify our rationale and address your concern, especially in light of the fact that we did not conduct a subgroup analysis due to the limited number of included studies. The reasons for including PD patients of different stages are written below:

Limited Number of Eligible Studies:

The primary reason for including studies with PD patients at various stages was the previous knowledge of the limited literature regarding PT in PD patients. Our systematic review and meta-analysis comprised only four articles, which precluded the possibility of conducting a meaningful subgroup analysis based on disease stage. Given this constraint, excluding studies based on disease stage would have further reduced dataset, potentially leading to an incomplete or biased synthesis of the evidence on the effectiveness of power training (PT).

Comprehensive Synthesis of Available Evidence:

By including studies across different stages of PD, our objective was to provide a comprehensive synthesis of the available evidence on PT’s impact on physical functional performance (PFP) in PD patients. Although the stage of disease may influence the response to exercise interventions, including a broader range of studies allows for a more holistic understanding of PT’s potential benefits across the disease spectrum. This approach is especially important in light of the limited research available on PT in PD.

Relevance to Clinical Practice:

Exercise-based interventions, including PT, are commonly implemented in clinical practice for PD patients regardless of their disease stage. Our inclusion criteria reflect the real-world scenario where PT is prescribed to patients at various stages of PD. While we acknowledge that the response to PT may differ depending on disease severity, our aim was to present findings that are relevant and applicable to a broad patient population. This approach ensures that our conclusions are more generalizable to the diverse range of patients seen in clinical settings.

Existing Literature on Exercise in PD:

Current literature supports the notion that exercise can be beneficial across different stages of PD. By including studies with different stages, our review aligns with the broader understanding that exercise is a crucial component of managing PD at any stage, and that PT may offer benefits regardless of disease severity.

In conclusion, while we acknowledge the influence of disease stage on exercise outcomes, the inclusion of studies with patients at different stages was necessary to maximize the available evidence and provide a comprehensive overview of PT’s effects on PFP in PD patients. We believe this approach, despite its limitations, contributes valuable insights that can inform both research and clinical practice.

We hope this answer could address your concerns and provide clarity on our methodological decisions.

*Intervention: The intervention description conflicts with the protocol registered in Prospero (Protocol number CRD42019139446). The types of exercises and equipment used for training differed among the included studies, as well as the muscle groups involved.*

Response: All included studies meet the criteria set out in the Prospero registry. The eligibility criteria (both inclusion and exclusion) set out in the registry were respected at the time of study selection. In order to present these criteria in the manuscript exactly as they are in the registered protocol, the following sentence was inserted into the revised version of the article (page 6, lines 117-124):

(I) interventions: power training is defined as a form of resistance training designed to enhance muscle power (force × velocity) by performing exercises with maximal concentric speed. The types of exercises categorized as power training in the included studies typically involved multi-joint movements, such as leg presses, chest presses, and knee extensions, utilizing equipment like free weights, machines, or resistance bands. All programs, regardless of intensity, frequency, or session duration, required participants to lift the weight 'as fast as possible' during the concentric phase. This approach is aimed at optimizing neuromuscular adaptations to improve power output in individuals with Parkinson's disease.

*Control: The use of comparators, control without intervention, and the control being other forms of physical activity or exercise (e.g., balance training, resistance training, aerobic exercise, Tai Chi, movement strategy training, fall prevention education, and home exercise prescription) could potentially introduce a confounding factor, as the speed of exercise execution is not consistently specified in the method descriptions of the articles included in this SR. In the study by Meng Ni et al. (2016), the control group had training characteristics resembling power training. The authors demonstrated that High-Speed yoga improves power similarly to Power Training. Could this study not be a confounding factor considering the objective of the systematic review under analysis? The study by Meng Ni et al. (2016) is referenced throughout the manuscript as being from 2015.*

Response: We thank the reviewer for the detailed and thoughtful question regarding the control groups used in our systematic review and meta-analysis, particularly concerning the potential confounding effects introduced by the variability in exercise interventions across the included studies.

Addressing the Confounding Factor:

Heterogeneity in Control Interventions:

We acknowledge the concern that the use of various forms of physical activity or exercise as comparators, such as balance training, resistance training, aerobic exercise, and others, could introduce heterogeneity and potentially confound the results. The inclusion of these diverse interventions reflects the reality of the current literature, where studies often employ different types of active controls to investigate the effects of specific exercise modalities like power training (PT).

Rationale for Inclusion Despite Potential Confounding:

Despite the potential confounding factors, the study by Meng Ni et al. (2016) was included in our review to provide a comprehensive overview of the current evidence on PT's effectiveness. The inclusion of this study, along with others that utilized different control interventions, allowed us to capture a broader range of comparisons and better understand PT's relative effectiveness. However, we recognize that this introduces heterogeneity, and we have explicitly acknowledged this limitation in our discussion.

Correction of the Citation Year:

We also appreciate the correction regarding the citation of Meng Ni et al. (2016) being referenced as a 2015 study throughout the manuscript. This will be corrected in the final version to ensure accuracy.

Conclusion:

In summary, while the inclusion of studies like Meng Ni et al. (2016) presents challenges due to the similarities in training characteristics with PT, their inclusion was necessary to provide a comprehensive synthesis of the available evidence. We acknowledge the potential confounding effects and have addressed these in our discussion, ensuring that the limitations are clearly communicated.

We hope this response adequately addresses your concerns, and we will ensure that the final manuscript reflects these considerations appropriately.

*Outcome: Authors must clearly define what was considered Functional Physical Performance (DFP), since the protocols listed for the primary outcome do not represent, in isolation, functional capacity.*

Response: We totally agree with the reviewer. Thank you for your feedback regarding the definition of Functional Physical Performance (FPP). I would like to highlight that the revised version of our manuscript now includes a clear and comprehensive definition of FPP in the introduction section (pages 3 and 4, lines 62 to 72). We have specifically detailed how it encompasses various domains such as strength, balance, mobility, and endurance, and how these are assessed through the selected protocols. These additions were made to ensure that the concept of FPP is fully articulated and that the rationale for the inclusion of each test is clear and well-justified. We believe these revisions address your concerns, and we appreciate your input in helping us improve our manuscript.

*Results*

*Considering the characteristics of the RCT interventions included in this SR, I understand that a meta-analysis would not be appropriate.*

We acknowledge the reviewer’s concern regarding the appropriateness of conducting a meta-analysis with only four randomized controlled trials (RCTs). Despite the limited number of studies, we believe that performing a meta-analysis is justified for the following reasons:

Synthesis of Evidence: Meta-analysis, even with a small number of studies, provides a more precise estimate of the intervention’s effect than individual studies. In our case, the included RCTs evaluated similar interventions — power training aimed at improving physical functional performance in patients with Parkinson’s disease — allowing for meaningful synthesis.

Heterogeneity Management: We employed a random-effects model to account for potential heterogeneity between the studies. Despite some variability in training protocols and outcome measures, the model assumes the studies are estimating different but related effects, ensuring that the results are robust and clinically relevant.

Given these considerations, we maintain that the meta-analysis offers valuable insights, providing a comprehensive assessment of the effect of power training in this population.

*Why did the authors not use the Risk of Bias 2 (ROB 2) risk of bias analysis tool, which is better suited for SR’s of RCT’s that are not physical therapy interventions.*

Response: We thank the reviewer. We now included the ROB II to evaluate the risk of bias. The information is added in the methods section (pages 8 and 9, lines 184 to 202) and in the results section (pages 23 and 24, lines 367 to 386) and figure 7.

The methodological quality of the included trials was evaluated using the Revised Cochrane risk-of-bias tool for randomized trials (RoB 2) [23]. This tool assesses bias across five key domains: 1) bias arising from the randomization process, 2) bias due to deviations from intended interventions, considering both the effect of assignment to the intervention and adherence to it, 3) bias due to missing outcome data, 4) bias in the measurement of the outcome, and 5) bias in the selection of the reported results. Each domain is examined through 3–7 signaling questions, with possible responses including: yes, probably yes, probably no, no, and no information. Based on the responses to these questions, an algorithm determines the risk of bias for each domain, categorizing it as “low risk of bias,” “high risk of bias,” or “some concerns.”

After evaluating each domain individually, an overall risk of bias judgment for each study was made using the same classification criteria. A study was deemed to have a “low risk of bias” if all domains met the required standards. If at least one domain raised “some concerns” or lacked clarity, the study was classified under “some concerns.” A “high risk of bias” designation was assigned when at least one domain did not meet the standards, or when multiple domains were classified as having “some concerns.” Two independent reviewers (SB and DP) assessed the risk of bias of the included studies, and any disagreements in the scoring of trials were resolved with a third reviewer.

The summary of the risk of bias analysis is depicted in Fig 7, according to the RoB2 tool [24]. Overall, the studies exhibited varying levels of risk. Two studies [12,14] demonstrated a high risk of bias across multiple domains, particularly in domains related to randomization and measurement of the outcome (D1 and D3). One study [15] showed some concerns, primarily in domain 5, related to the appropriateness of the analysis considering the missing data and deviations from the intended intervention. Another study [13] displayed a mix of low risk in randomization but concerns or high risks in other areas, especially in domain 4, which relates to the influence of missing data on the outcomes.

The high risk in domain 1 for some studies [12,15] was attributed to the lack of clarity on the randomization process. Similarly, domain 3 had concerns due to potential deviations from the intended interventions that were not accounted for in the analysis [12,14]. Domain 5, concerning selective reporting of outcomes, also contributed to the overall concerns as it was unclear whether the outcomes were analyzed as per the pre-specified plans [13]. These issues collectively led to an overall categorization of high risk for some studies and some concerns for others.

*Fig. 7 Summary of risk of bias by domain and study.*

*Discussion*

*The authors note that "The results of the present study may partially be explained by some factors. First, some studies showed high heterogeneity – ranging from 88% for PWS and 83% for FWS". These two parameters among the seven analyzed show a high coefficient of heterogeneity; for the other studies, there was no heterogeneity (i2 = 0). Additionally, the authors highlight a range of factors that may explain the analysis results, such as variations in the training protocols of the included studies and the involved muscle groups.*

*Why analyze studies with such diverse exercise doses? The discussion of the results of this SR is compromised due to the various limitations that compromise the meta-analysis of the data. Such limitations are presented by the authors in the discussion section.*

*The discussion is more focused on explaining the factors that compromise the meta-analysis than its potential results. This is because there is no room for a meta-analysis considering the studies included.*

Response: We thank the reviewer for the detailed and constructive feedback on our manuscript. We appreciate the opportunity to clarify our rationale and address your concerns regarding the heterogeneity and the diversity of exercise doses in the studies included in our meta-analysis.

Justification for Analyzing Studies with Diverse Exercise Doses

The inclusion of studies with varying exercise doses and training protocols was a necessary decision to address the current state of the literature on power training (PT) in Parkinson's Disease (PD). The field is still evolving, and there is a lack of standardized protocols across studies. This variability reflects the real-world challenges faced by clinicians and researchers when implementing PT interventions in PD populations. We aimed to provide a comprehensive synthesis of the available evidence, despite the inherent heterogeneity.

Addressing Heterogeneity in the Meta-Analysis

We acknowledge that the heterogeneity observed in parameters such as Preferred Walking Speed (PWS) and Fast Walking Speed (FWS) is high (I² = 88% and 83%, respectively). This level of heterogeneity is not uncommon in meta-analyses that include interventions with varied protocols, especially in emerging fields where standardization is limited. The presence of heterogeneity underscores the need for more rigorous and standardized future research, which is a key recommendation of our study.

However, it is important to note that the absence of heterogeneity (I² = 0) in other outcomes does not negate the value of the meta-analysis. In fact, it highlights that, despite the diversity in exercise doses and protocols, there are aspects of physical functional performance that may respond consistently to PT across different studies. These findings are crucial for identifying robust outcomes that can be targeted in clinical practice.

Discussion Focus

We recognize that our discussion may have heavily emphasized the limitations and factors contributing to heterogeneity, potentially overshadowing the interpretation of the meta-analysis results. This was done to ensure transparency and to contextualize our findings within the broader scope of the current literature. However, we agree that a balanced discussion should also highlight the meaningful results obtained despite these limitations.

To address this, we revised the discussion to better integrate the potential clinical implications of our findings, emphasizing the positive outcomes observed in the meta-analysis. We also elaborated on how the consistent results in some parameters, despite the varied exercise doses, contribute valuable insights into the effects of PT in PD. The following text was included in the discussion section (page 28, lines 471 to 482):

While our discussion highlighted several factors that may have compromised the meta-analysis, it is important to also focus on the potential clinical implications of the results. The consistent improvements seen in key parameters, such as TUG and BBA, suggest that PT can have significant benefits in improving PFP in patients with PD. These findings are particularly relevant in enhancing mobility and balance, which are crucial for maintaining independence in daily activities [46]. The variability in exercise dosages across the included studies may reflect real-world differences in clinical practice, yet the consistent positive outcomes observed in some measures underscore the adaptability and effectiveness of PT interventions. Therefore, while methodological limitations exist, the results of this meta-analysis contribute valuable insights into the role of PT as a therapeutic strategy in PD, demonstrating its potential to mitigate functional decline and improve quality of life.

In conclusion, while the heterogeneity and diversity of the included studies present challenges, they also provide a realistic reflection of the current state of research in this area. By synthesizing this evidence, our meta-analysis offers important insights that can guide future research and clinical practice. We appreciate your feedback and will ensure that the revised discussion more clearly balances the interpretation of the meta-analysis results with the consideration of its limitations.

Thank you for helping us improve our manuscript.

*Reviewer #3: I am grateful for the opportunity to provide feedback on the manuscript entitled "EFFECT OF POWER TRAINING ON PHYSICAL FUNCTIONAL PERFORMANCE OF PATIENTS WITH PARKINSON’S DISEASE: A SYSTEMATIC REVIEW AND METAANALYSIS OF RANDOMIZED CONTROLLED TRIALS" submitted to PLOS One. Overall, the manuscript is well-crafted; however, several points warrant consideration.*

*1- Please elucidate the concept of power training within the "Intervention" component of your Population, Intervention, Comparison, Outcome (PICO) framework. Specifically, clarify the types of exercises categorized as power training within the context of your systematic review.*

Response: Thank you for your insightful comment. To address your request, we have clarified the concept of power training in the "Intervention" component of our PICO framework. Specifically, within the context of this systematic review, power training is defined as a form of resistance training that emphasizes performing exercises with maximal concentric speed, aiming to enhance muscle power (force × velocity). The types of exercises categorized as power training in the included studies typically involved multi-joint movements, such as leg presses, chest presses, and knee extensions, often utilizing free weights, machines, or resistance bands. These exercises were designed to be performed with submaximal loads, lifted 'as fast as possible' during the concentric phase of the movement. This method is intended to optimize the neuromuscular adaptations necessary for improving power output in individuals with Parkinson’s disease. We have incorporated this clarification into the "Intervention" section of the manuscript for improved understanding. Aiming to improve this concept in the new version of the manuscript the text was adjusted as follows (page 6, lines 117 to 124):

Power training is defined as a form of resistance training designed to enhance muscle power (force × velocity) by performing exercises with maximal concentric speed. The types of exercises categorized as power training in the included studies typically involved multi-joint movements, such as leg presses, chest presses, and knee extensions, utilizing equipment like free weights, machines, or resistance bands. All programs, regardless of intensity, frequency, or session duration, required participants to lift the weight 'as fast as possible' during the concentric phase. This approach is aimed at optimizing neuromuscular adaptations to improve power output in individuals with Parkinson's disease.

*2- The rationale behind the authors' selection of Hedges' g over Cohen's d as the preferred effect size measure is not evident. Justification for this choice is necessary to enhance the clarity and rigor of the methodology.*

Response: Choosing between Hedges' g and Cohen's d in meta-analysis depends on the context of the studies being analyzed. Both are measures of effect size, but there are key reasons why Hedges' g is often preferred over Cohen's d, especially in meta-analysis:

1. Correction for Small Sample Sizes: Hedges' g includes a correction factor to account for the bias that small sample sizes can introduce in effect size estimates. This correction helps to provide a more accurate estimate of the population effect size, making Hedges' g particularly useful when the studies included in the meta-analysis have small sample sizes.
2. Unbiased Estimate: Hedges' g is designed to provide an unbiased estimate of the population effect size by adjusting Cohen's d. This adjustment is especially important when synthesizing results from studies with varying sample sizes, ensuring that the effect size estimates are not disproportionately influenced by smaller studies.
3. Consistency Across Studies: By using Hedges' g, researchers ensure consistency in effect size estimates across studies, as it provides a more reliable measure when sample sizes are small. This consistency is crucial for meta-analysis, which combines results from multiple studies to draw overall conclusions.
4. Standardization: Both Hedges' g and Cohen's d are standardized measures of effect size, but Hedges' g's adjustment makes it more appropriate for meta-analytic contexts where studies may differ in sample size and precision.

While Cohen's d is a useful measure in individual studies, Hedges' g is generally favored in meta-analysis because it provides a more accurate and unbiased estimate of the overall effect size, particularly when dealing with a range of sample sizes across different studies.

References:

1) Lipsey, M. W., & Wilson, D. B. (2001). Practical meta-analysis. SAGE publications, Inc.
2) Hedges, L. V., & Olkin, I. (1985). Statistical Methods for Meta-Analysis. Academic Press.
3) Cohen, J. (1988). Statistical Power Analysis for the Behavioral Sciences (2nd ed.). Lawrence Erlbaum Associates.

Aiming to clarify the justification for the use of Hedges' g, we added the sentence below in the statistical analysis section of the new version of the manuscript (page 10, lines 212 to 215):

"Hedges' g includes a correction for small sample size bias and is often recommended in meta-analyses because correcting for small sample sizes results in a more conservative and precise estimate, especially when the meta-analysis includes studies with varying sample sizes."

*3- I recommend employing the Cochrane Version 2 Risk of Bias (RoB 2) tool instead of the PEDro scale. The RoB 2 tool is specifically designed for assessing the risk of bias in interventional studies, aligning more closely with the objectives of this review.*

Response: We appreciate the reviewer comments. We now included the ROB II to evaluate the risk of bias. The information is added in the methods section (pages 8 and 9, lines 184 to 202) and in the results section (pages 23 and 24, lines 367 to 386) and figure 7.

The methodological quality of the included trials was evaluated using the Revised Cochrane risk-of-bias tool for randomized trials (RoB 2) [23]. This tool assesses bias across five key domains: 1) bias arising from the randomization process, 2) bias due to deviations from intended interventions, considering both the effect of assignment to the intervention and adherence to it, 3) bias due to missing outcome data, 4) bias in the measurement of the outcome, and 5) bias in the selection of the reported results. Each domain is examined through 3–7 signaling questions, with possible responses including: yes, probably yes, probably no, no, and no information. Based on the responses to these questions, an algorithm determines the risk of bias for each domain, categorizing it as “low risk of bias,” “high risk of bias,” or “some concerns.”

After evaluating each domain individually, an overall risk of bias judgment for each study was made using the same classification criteria. A study was deemed to have a “low risk of bias” if all domains met the required standards. If at least one domain raised “some concerns” or lacked clarity, the study was classified under “some concerns.” A “high risk of bias” designation was assigned when at least one domain did not meet the standards, or when multiple domains were classified as having “some concerns.” Two independent reviewers (SB and DP) assessed the risk of bias of the included studies, and any disagreements in the scoring of trials were resolved with a third reviewer.

The summary of the risk of bias analysis is depicted in Fig 7, according to the RoB2 tool [24]. Overall, the studies exhibited varying levels of risk. Two studies [12,14] demonstrated a high risk of bias across multiple domains, particularly in domains related to randomization and measurement of the outcome (D1 and D3). One study [15] showed some concerns, primarily in domain 5, related to the appropriateness of the analysis considering the missing data and deviations from the intended intervention. Another study [13] displayed a mix of low risk in randomization but concerns or high risks in other areas, especially in domain 4, which relates to the influence of missing data on the outcomes.

The high risk in domain 1 for some studies [12,15] was attributed to the lack of clarity on the randomization process. Similarly, domain 3 had concerns due to potential deviations from the intended interventions that were not accounted for in the analysis [12,14]. Domain 5, concerning selective reporting of outcomes, also contributed to the overall concerns as it was unclear whether the outcomes were analyzed as per the pre-specified plans [13]. These issues collectively led to an overall categorization of high risk for some studies and some concerns for others.

*Fig. 7 Summary of risk of bias by domain and study.*

*4- Given the systematic nature of this review involving interventional studies, it is imperative for the authors to evaluate the certainty of evidence using the Grading of Recommendations Assessment, Development, and Evaluation (GRADE) approach.*

Response: We thank the reviewer for the comment. We included the GRADE approach. The information is added in the methods section (page 8, lines 172 to 182) and in the result section (pages 20 to 23, lines 337 to 365) and table 3.

*5- The methodology utilized in the random effects model, such as the DerSimonian-Laird (DL) or Restricted Maximum Likelihood (REML) method, should be clearly specified.*

Response: We thank the reviewer for this recommendation. We added this information on the “Statistical Analysis” Section (page 10, lines 208 to 212), as you can see below:

“The Hedges’ g effect sizes were presented as standardized mean differences (SMD - a measure of effect, recommended to be used when the study reports the effectiveness of an intervention in continuous measurements, especially in cases of different measurement methods) and calculations were performed using random-effect models, applying the DerSimonian-Laird method.”

*6- In the forest plots, it is essential to denote which side favors power training and which side favors the control treatment.*

Response: We thank the reviewer for this comment. We included the information requested in all the forest plots (figures 2 to 7).

*7- Notably, there is a deficiency in the manuscript's discussion regarding potential mechanisms underlying considerable statistical heterogeneity observed among the results.*

Response: We totally agree with the reviewer. We included in the limitations section more possible explanations for the considerable statistical heterogeneity observed among the results (pages 28 and 29, lines 491 to 510).

*6. PLOS authors have the option to publish the peer review history of their article (what does this mean?). If published, this will include your full peer review and any attached files.*

Response: We have no opposition to include the peer review history.
